# Association of inflammatory cytokines with lung function, chronic lung diseases, and COVID-19

**DOI:** 10.1016/j.isci.2024.110704

**Published:** 2024-08-09

**Authors:** Marina O. Rontogianni, Dipender Gill, Emmanouil Bouras, Alexandros-Georgios Asimakopoulos, Ioanna Tzoulaki, Ville Karhunen, Terho Lehtimäki, Olli Raitakari, Matthias Wielscher, Veikko Salomaa, Sirpa Jalkanen, Marko Salmi, Markku Timonen, James Yarmolinsky, Jing Chen, Martin D. Tobin, Abril G. Izquierdo, Karl-Heinz Herzig, Anne E. Ioannides, Marjo-Riitta Jarvelin, Abbas Dehghan, Konstantinos K. Tsilidis

**Affiliations:** 1 Department of Hygiene and Epidemiology, University of Ioannina School of Medicine, Ioannina, Greece; 2 Department of Epidemiology and Biostatistics, School of Public Health, Imperial College London, London, UK; 3 Research Unit of Population Health, Faculty of Medicine, University of Oulu, Oulu, Finland; 4 Research Unit of Mathematical Sciences, University of Oulu, Oulu, Finland; 5 Department of Clinical Chemistry, Faculty of Medicine & Health Technology, Tampere University, Tampere, Finland; 6 Finnish Cardiovascular Research Center Tampere, Faculty of Medicine & Health Technology, Tampere University, Tampere, Finland; 7 Fimlab Laboratories, Tampere, Finland; 8 Centre for Population Health Research, University of Turku and Turku University Hospital, Turku, Finland; 9 Research Centre of Applied and Preventive Cardiovascular Medicine, University of Turku, Turku, Finland; 10 Department of Clinical Physiology and Nuclear Medicine, Turku University Hospital, Turku, Finland; 11 Finnish Institute for Health and Welfare, Helsinki, Finland; 12 MediCity Research Laboratory, University of Turku, Turku, Finland; 13 Institute of Biomedicine, University of Turku, Turku, Finland; 14 InFLAMES Fiagship, University of Turku, Turku, Finland; 15 Research Unit of Population Health, University of Oulu, Oulu, Finland; 16 Medical Research Center (MRC) and University Hospital, Oulu, Finland; 17 MRC Integrative Epidemiology Unit, University of Bristol, Bristol, UK; 18 Population Health Sciences, Bristol Medical School, University of Bristol, Bristol, UK; 19 Genetic Epidemiology Group, Department of Health Sciences, University of Leicester, Leicester, UK; 20 National Institute for Health Research, Leicester Respiratory Biomedical Research Centre, Glenfield Hospital, Leicester, UK; 21 Department of Health Sciences, University of Leicester, Leicester, UK; 22 Research Unit of Biomedicine and Internal Medicine, Faculty of Medicine, University of Oulu, Oulu, Finland; 23 Pediatric Gastroenterology and Metabolic Diseases, Pediatric Institute, Poznan University of Medical Sciences, Poznan, Poland; 24 Department of Primary Care and Public Health, School of Public Health, Imperial College London, White City Campus, London, UK; 25 Unit of Primary Care, Oulu University Hospital, Oulu, Finland; 26 Department of Life Sciences, College of Health and Life Sciences, Brunel University London, Uxbridge, UK; 27 Cancer Epidemiology Unit, Nuffield Department of Population Health, University of Oxford, Oxford, UK; 28 UK Dementia Research Institute at Imperial College London, London, UK

**Keywords:** Health sciences, Respiratory medicine, Disease, Association analysis

## Abstract

We investigated the effects of 35 inflammatory cytokines on respiratory outcomes, including COVID-19, asthma (atopic and non-atopic), chronic obstructive pulmonary disease (COPD), and pulmonary function indices, using Mendelian randomization and colocalization analyses. The emerging associations were further explored using observational analyses in the UK Biobank. We found an inverse association between genetically predicted macrophage colony stimulating factor (MCSF), soluble intercellular adhesion molecule-1 (sICAM), and soluble vascular cell adhesion molecule-1 with risk of COVID-19 outcomes. sICAM was positively associated with atopic asthma risk, whereas tumor necrosis factor-alfa showed an inverse association. A positive association was shown between interleukin-18 and COPD risk (replicated in observational analysis), whereas an inverse association was shown for interleukin-1 receptor antagonist (IL-1ra). IL-1ra and monocyte chemotactic protein-3 were positively associated with lung function indices, whereas inverse associations were shown for MCSF and interleukin-18 (replicated in observational analysis). Our results point to these cytokines as potential pharmacological targets for respiratory traits.

## Highlights

• We assessed the effects of 35 unique cytokines on respiratory outcomes
• Mendelian randomization and colocalization analyses were performed
• The emerging associations were further explored using observational analyses
• Our results implicate several cytokines in respiratory disease risk

## Graphical abstract

**Figure:**
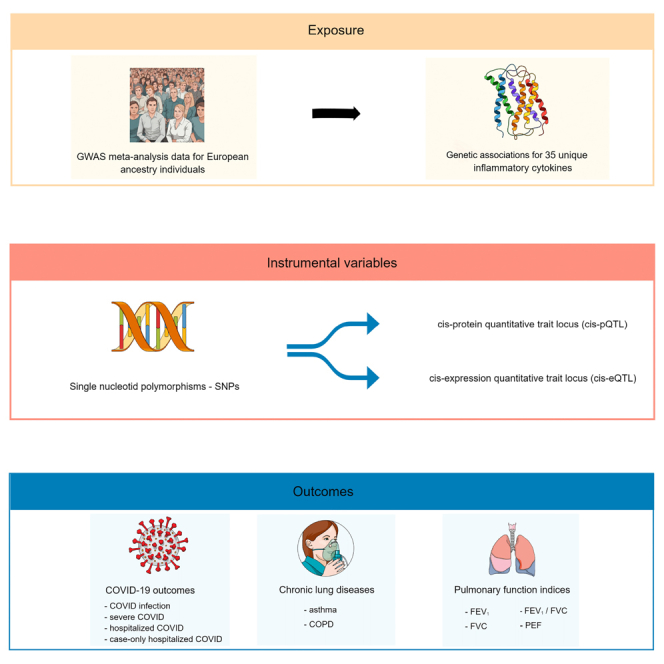

## Introduction

Chronic diseases of the respiratory system, including asthma and chronic obstructive pulmonary disease (COPD), represent some of the most common causes of morbidity and mortality worldwide.^1^ In 2017, approximately 545 million people were living with chronic respiratory disease globally and this accounted for 3.9 million deaths.^2^ Impairment of lung function, as measured by forced expiratory volume in the first second of exhalation (FEV_1_), forced vital capacity (FVC) and peak expiratory flow rate (PEF), is a predictor of adverse health outcomes in respiratory disease, as well as in all-cause mortality.^3^ The importance of respiratory health has been highlighted by the coronavirus disease 2019 (COVID-19) pandemic, which accounted for approximately 18 million excess deaths by the end of 2021.^4^ Activation of inflammatory pathways plays a crucial role in the pathophysiology of both COVID-19^5^ and chronic respiratory diseases.^6^ Even though inflammation is a physiological reaction to harmful stimuli, in case of immune dysregulation and hyperinflammation, i.e., a surge of inflammatory factors, it can be detrimental and even life-threatening. Characteristic is the paradigm of the cytokine release syndrome in COVID-19, for which several immunomodulatory drugs are under investigation or approved, such as tocilizumab (interleukin-6 (IL-6) receptor antagonist).^5^^,^^7^

Establishing the role of inflammatory cytokines in impaired lung function and respiratory disease is therefore of direct relevance to understanding the pathophysiology of these conditions, and thus identifying therapeutic opportunities. However, randomized controlled trials can be prohibitively expensive and time-consuming,^8^ and observational studies have the disadvantage of possible confounding or reverse causation bias. Mendelian randomization (MR) can overcome these limitations by leveraging germline genetic variants as instrumental variables for studying the effect of altering an exposure on a disease outcome.^9^ The random allocation of genetic variants at meiosis and conception means that their associations with disease outcomes are less likely to be subject to environmental confounding or reverse causation bias. Further, when investigating protein drug target genes,^10^ colocalization analysis can be used to explore whether any genetic associations are attributable to a common causal variant across protein targets and disease outcomes, rather than distinct variants that are in linkage disequilibrium (LD).^11^

In this study, we perform MR and colocalization analyses to investigate the effects of 35 unique inflammatory cytokines on FEV_1_, FVC, the FEV_1_/FVC ratio, PEF, and risks of asthma (atopic and non-atopic), COPD and COVID-19. Thus, we aim to offer advanced insight into the potential causal role of inflammatory cytokines on respiratory disease and thus identify pharmacological targets for prioritization in clinical studies.

Some of the results of this study have been accepted in the form of a conference abstract.^12^

## Results

### Instrument characteristics

We used summary genetic association estimates from a genome-wide association study (GWAS) meta-analysis of circulating levels of 47 inflammatory cytokines.^13^^,^^14^^,^^15^^,^^16^^,^^17^ Among them, instruments were found for 31 and 27 cytokines using the *cis*-protein quantitative trait locus (*cis*-pQTL) and *cis*-expression quantitative trait locus (*cis*-eQTL) definitions, respectively. These included 35 unique cytokines that are presented in Table S1. Of the 27 eQTL cytokines, 6 had weak instruments (F-statistic<10) and were excluded from the analysis, and one had no instruments after the clumping of the data, resulting in 20 eQTL cytokines that were included in the analysis.

### Cytokine associations with outcomes using Mendelian randomization

#### Coronavirus disease 2019 outcomes

**Figure 1:**
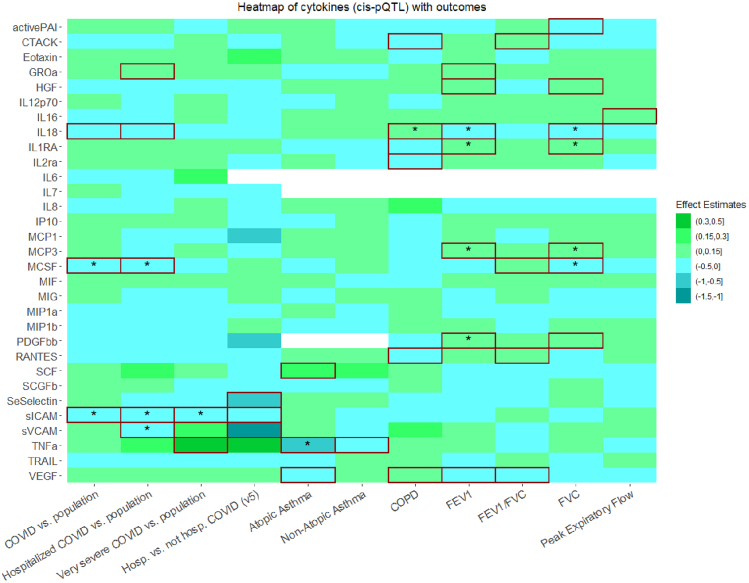
Heatmap of cytokines (*cis*-pQTL) with outcomes Summary of the Mendelian randomization results with the inverse variance weighted method based on the *cis*-pQTL instrument definition, where squared tiles indicate nominally significant associations (*p* < 0.05), the asterisk denotes that the association was significant when considering multiple comparison correction (false discovery rate <5%), and associations for which no instrument was available are presented as white tiles. Abbreviations: *cis*-pQTL, *cis*-protein quantitative trait locus; activePAI, active plasminogen activator inhibitor-1; CTACK, cutaneous T cell attracting chemokine; GROa, growth regulated oncogene-alpha; HGF, hepatocyte growth factor; IL-12p70, interleukin 12p70; IL 16, interleukin 16; IL 18, interleukin 18; IL1RA, interleukin 1 receptor antagonist; IL2ra, interleukin-2 receptor antagonist; IL6, interleukin 6; IL7, interleukin 7; IL8, interleukin 8; IP10, interferon gamma-induced protein 10; MCP1, monocyte chemotactic protein-1; MCP3, monocyte specific chemokine; MCSF, macrophage colony stimulating factor; MIF, macrophage migration inhibitory factor; MIG, monokine induced by interferon-gamma; MIP1a, macrophage inflammatory protein 1a; MIP1b, macrophage inflammatory protein 1b; PDGFbb, platelet-derived growth factor BB; RANTES, beta-chemokine RANTES; SCF, stem cell factor; SCGFb, stem cell growth factor beta; SeSelectin, soluble Eselectin; sICAM, soluble intercellular adhesion molecule 1; sVCAM, soluble vascular cell adhesion molecule 1; TNF a, tumor necrosis factor a; TRAIL, TNF-related apoptosis inducing ligand; VEGF, vascular endothelial growth factor; COPD, chronic obstructive pulmonary disease; FEV1, forced expiratory volume measured in the first second of exhalation; and FVC, forced vital capacity.

**Figure 2:**
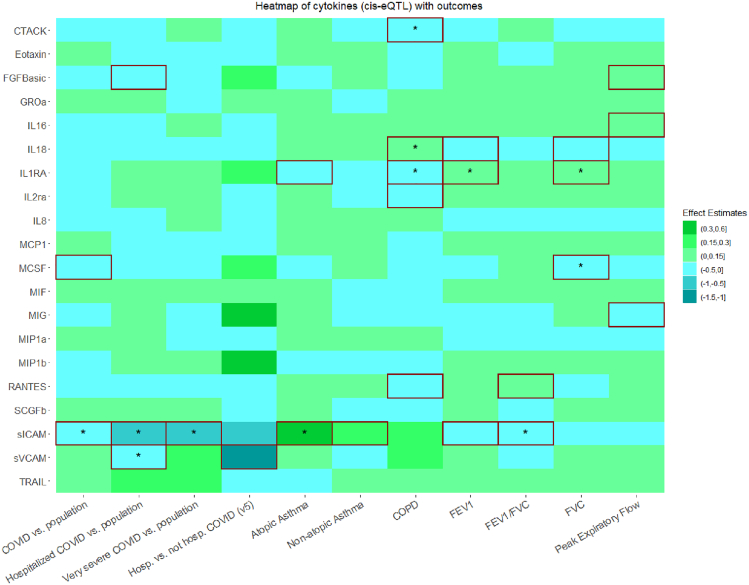
Heatmap of cytokines (*cis*-eQTL) with outcomes Summary of the Mendelian randomization results with the Inverse Variance Weighted method based on the *cis*-eQTL instrument definition, where squared tiles indicate nominally significant associations (*p* < 0.05), the asterisk denotes that the association was significant when considering multiple comparison correction (false discovery rate <5%), and associations for which no instrument was available are presented as white tiles. Abbreviations: *cis*-eQTL, *cis*-expression quantitative trait locus; CTACK, cutaneous T cell attracting chemokine; FGFBasic, basic fibroblast growth factor ; GROa, growth regulated oncogene-alpha; IL 16, interleukin 16; IL 18, interleukin 18; IL1RA, interleukin 1 receptor antagonist; IL2ra, interleukin-2 receptor antagonist; IL8, interleukin 8; MCP1, monocyte chemotactic protein-1; MCSF, macrophage colony stimulating factor; MIF, macrophage migration inhibitory factor; MIG, monokine induced by interferon-gamma; MIP1a, macrophage inflammatory protein 1a; MIP1b, macrophage inflammatory protein 1b; RANTES, beta-chemokine RANTES; SCGFb, stem cell growth factor beta; sICAM, soluble intercellular adhesion molecule 1; sVCAM, soluble vascular cell adhesion molecule 1; TRAIL, TNF-related apoptosis inducing ligand; COPD, chronic obstructive pulmonary disease; FEV1, forced expiratory volume measured in the first second of exhalation; and FVC, forced vital capacity.

**Figure 3:**
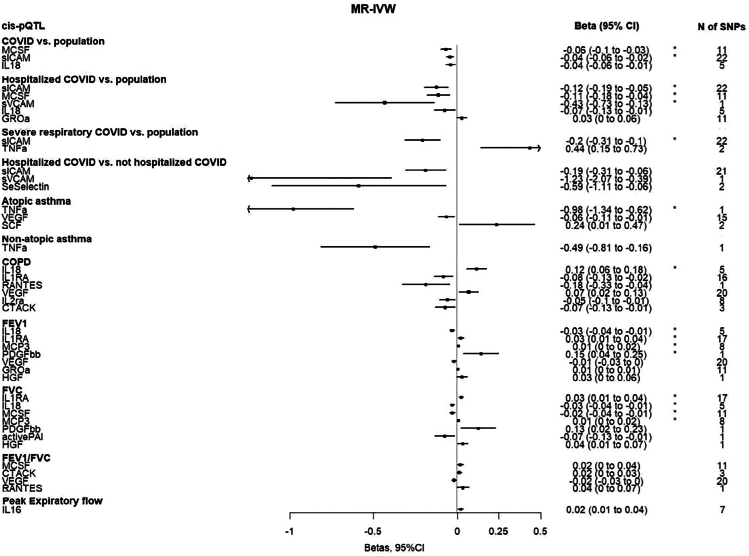
Forest plot showing the nominally significant (*p* < 0.05) MR-IVW estimates (beta, 95%CI) per standard deviation (SD) increase in the natural scale of each cytokine based on the pQTL definition The asterisk denotes that the association was significant when considering multiple comparison corrections (false discovery rate <5%). Abbreviations: MR-IVW, Mendelian randomization Inverse Variance Weighted method; *cis*-pQTL, *cis*-protein quantitative trait locus; CI, confidence interval; SNP, single nucleotide polymorphism; COPD, chronic obstructive pulmonary disease; FEV1, forced expiratory volume measured in the first second of exhalation; FVC, forced vital capacity; MCSF, macrophage colony stimulating factor; sICAM, soluble intercellular adhesion molecule 1; IL 18, interleukin 18; sVCAM, soluble vascular cell adhesion molecule 1; GROa, growth regulated oncogene-alpha; TNF a, tumor necrosis factor a; SeSelectin, soluble E-selectin; VEGF, vascular endothelial growth factor; SCF, stem cell factor; IL1RA, interleukin 1 receptor antagonist; RANTES, beta chemokine RANTES; IL2ra, interleukin-2 receptor antagonist; CTACK, cutaneous T cell attracting chemokine; MCP3, monocyte specific chemokine; PDGFbb, platelet-derived growth factor BB; HGF, hepatocyte growth factor; activePAI, active plasminogen activator inhibitor- 1; and IL 16, interleukin 16.

**Figure 4:**
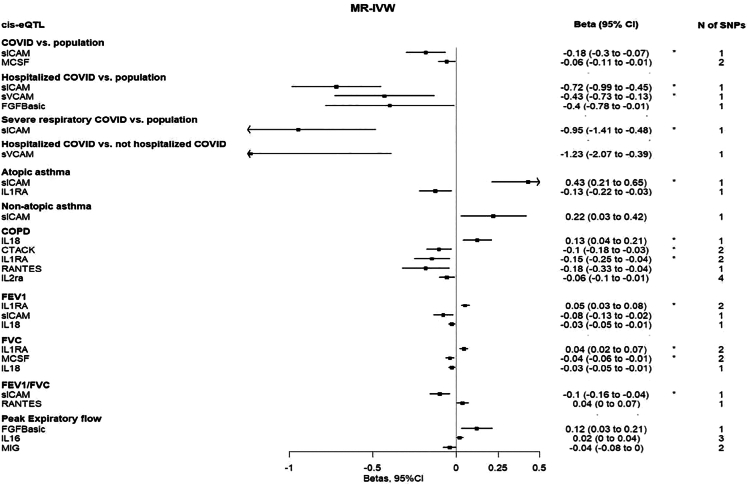
Forest plot showing the nominally significant (*p* < 0.05) MR-IVW estimates (beta, 95%CI) per standard deviation (SD) increase in the natural scale of each cytokine based on the eQTL definition The asterisk denotes that the association was significant when considering multiple comparison correction (false discovery rate <5%). Abbreviations: MR-IVW, Mendelian randomization Inverse Variance Weighted method; *cis*-eQTL, *cis*-expression quantitative trait locus; CI, confidence interval; SNP, single nucleotide polymorphism; COPD, chronic obstructive pulmonary disease; FEV1, forced expiratory volume measured in the first second of exhalation; FVC, forced vital capacity; sICAM, soluble intercellular adhesion molecule 1; MCSF, macrophage colony stimulating factor; sVCAM, soluble vascular cell adhesion molecule 1; FGFBasic, basic fibroblast growth factor ; IL1RA, interleukin 1 receptor antagonist; IL 18, interleukin 18; CTACK, cutaneous T cell attracting chemokine; RANTES, beta-chemokine RANTES; IL2ra, interleukin-2 receptor antagonist; IL 16, interleukin 16; and MIG, monokine induced by interferon-gamma.

**Figure 5:**
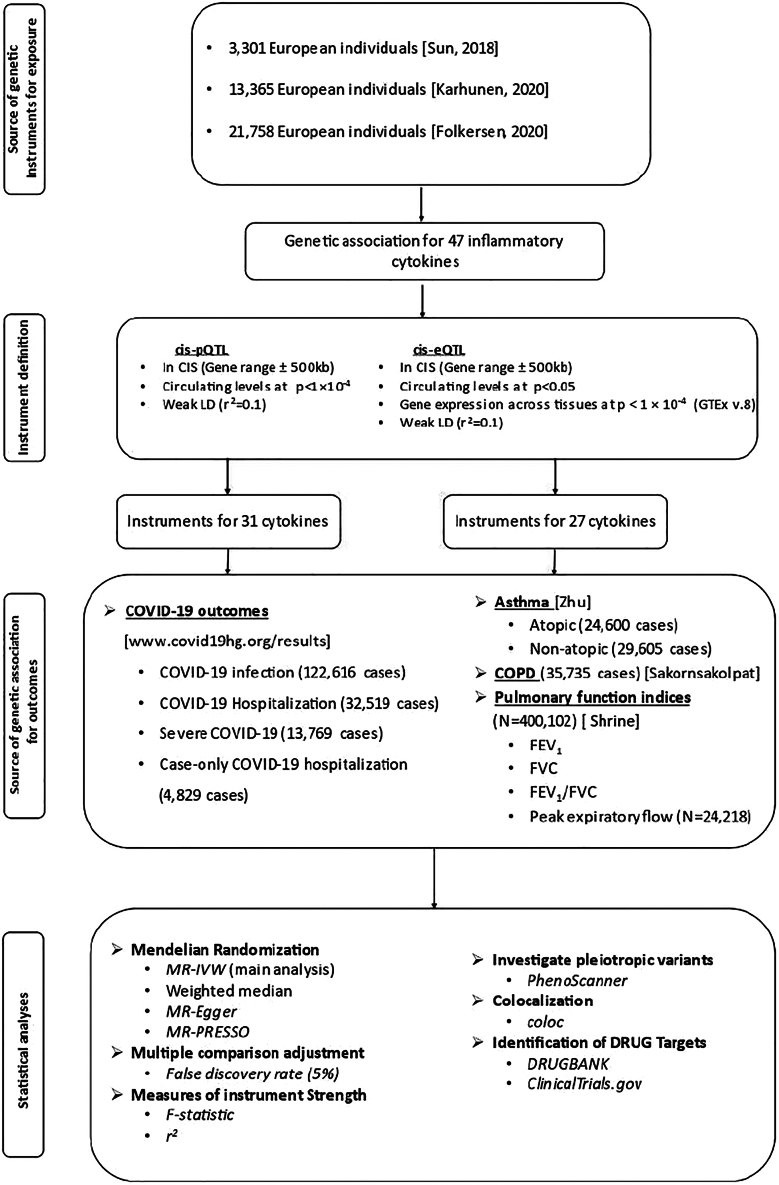
Analysis plan Abbreviations: *cis*-pQTL, *cis*-protein quantitative trait locus; *cis*-eQTL, *cis*-expression quantitative trait locus; LD, linkage disequilibrium; GTEx, Genotype-Tissue Expression database; COPD, chronic obstructive pulmonary disease; FEV1, forced expiratory volume measured in the first second of exhalation; FVC, forced vital capacity; and MR-IVW, Mendelian randomization Inverse Variance Weighted method.

Using the *cis*-pQTL instrument definition and considering a false discovery rate (FDR) < 5%, genetically proxied concentrations of macrophage colony stimulating factor (MCSF) were inversely associated with the risks of SARS-CoV-2 infection and hospitalization due to COVID-19 (odds ratio [OR]: 0.94, 95% confidence interval [CI]: 0.91 to 0.97, p (not FDR corrected) = 1.0 × 10^−4^ and OR: 0.90, 95%CI: 0.84 to 0.96, *p* = 1.4 × 10^−3^, respectively). Genetically proxied concentrations of soluble intercellular adhesion molecule 1 (sICAM) were inversely associated with the risk of SARS-CoV-2 infection (OR: 0.96, 95%CI: 0.94 to 0.98, *p* = 3.0 × 10^−4^), very severe respiratory confirmed COVID-19 (OR: 0.82, 95%CI: 0.73 to 0.91, *p* = 1.0 × 10^−4^) and hospitalization due to COVID-19 (OR: 0.89, 95%CI: 0.82 to 0.95, *p* = 8.0 × 10^−4^); hospitalization due to COVID-19 was also inversely associated with soluble vascular cell adhesion molecule 1 (sVCAM) (OR: 0.65, 95%CI: 0.48 to 0.88, *p* = 4.5 × 10^−3^). Using the *cis*-eQTL criteria, similar results were found for sICAM (OR: 0.83, 95%CI: 0.74 to 0.93, *p* = 2.0 × 10^−3^ for SARS-CoV-2 infection; OR: 0.49, 95%CI: 0.37 to 0.64, *p* = 1.26 × 10^−7^ for hospitalization due to COVID-19; OR: 0.39, 95%CI: 0.24 to 0.62, *p* = 1.0 × 10^−4^ for very severe COVID-19), and for sVCAM (OR: 0.65, 95%CI: 0.48 to 0.88, *p* = 4.5 × 10^−3^ for hospitalization due to COVID-19) (Figures 1, 2, 3, 4, and 5).

#### Atopic and non-atopic asthma

Using the *cis*-pQTL instrument definition, an inverse association was found between genetically proxied concentrations of tumor necrosis factor-alfa (TNF-a) and risk of atopic asthma (OR: 0.38, 95%CI: 0.26 to 0.54, *p* = 9.78 × 10^−8^) (FDR<5%). Using the eQTL criteria, an FDR significant positive association was found for sICAM (OR: 1.54, 95%CI: 1.24 to 1.91, *p* = 1.0 × 10^−4^). Using either the *cis*-pQTL or the *cis*-eQTL instrument definition, no FDR significant association was found with non-atopic asthma (Figures 1, 2, 3, and 4).

#### Chronic obstructive pulmonary disease

Using the *cis*-pQTL definition, the risk of COPD was higher with increased genetically proxied concentrations of interleukin-18 (IL-18) (OR: 1.13, 95%CI: 1.06 to 1.20, *p* = 1.0 × 10^−4^) (FDR<5%). Using the eQTL criteria, the positive and FDR significant association for IL-18 was replicated (OR: 1.13, 95%CI: 1.04 to 1.24, *p* = 4.2 × 10^−3^), and additional inverse FDR significant associations were shown for interleukin-1 receptor antagonist (IL-1ra) (OR: 0.86, 95%CI: 0.78 to 0.96, *p* = 7.0 × 10^−3^) and cutaneous T cell attracting chemokine (CTACK) (OR: 0.90, 95%CI: 0.84 to 0.97, *p* = 5.8 × 10^−3^) (Figures 1, 2, 3, and 4).

#### Forced expiratory volume in the first second of exhalation

Using the *cis*-pQTL instrument definition criteria and considering an FDR<5%, a positive association was found between IL-1ra (beta: 0.03, 95%CI: 0.01 to 0.04, *p* = 3.4 × 10^−3^), monocyte chemotactic protein-3 (MCP3) (0.01, 95%CI: 0.00 to 0.02, *p* = 4.7 × 10^−3^), platelet derived growth factor-BB (PDGFbb) (0.15, 95%CI: 0.04 to 0.25, *p* = 6.0 × 10^−3^) and FEV_1_, whereas a negative association was found for IL-18 (−0.03, 95%CI: −0.04 to −0.01, *p* = 2.0 × 10^−4^). Using the eQTL criteria, the positive and FDR significant association of IL-1ra was replicated (0.05, 95%CI: 0.03 to 0.08, *p* = 3.7 × 10^−5^) (Figures 1, 2, 3, and 4).

#### Forced vital capacity

Using the *cis*-pQTL definition, we found evidence (FDR<5%) of a positive association between genetically proxied concentrations of IL-1ra (beta: 0.03, 95%CI: 0.01 to 0.04, *p* = 1.0 × 10^−4^), MCP3 (0.01, 95%CI: 0.00 to 0.02, *p* = 2.6 × 10^−3^) and FVC; negative associations were found for IL-18 (−0.03, 95%CI:-0.04 to −0.01, *p* = 5.0 × 10^−4^) and MCSF (−0.02, 95%CI: −0.04 to −0.01, *p* = 2.3 × 10^−3^). Using the eQTL criteria, similar FDR significant associations were found for IL-1ra (0.04, 95%CI: 0.02 to 0.07, *p* = 5.0 × 10^−4^) and MCSF (−0.04, 95%CI: −0.06 to −0.01, *p* = 3.6 × 10^−3^) (Figures 1, 2, 3, and 4).

#### Forced expiratory volume in the first second of exhalation/forced vital capacity

Using the *cis*-pQTL criteria, no FDR significant association was found between genetically proxied concentrations of cytokines and FEV_1_/FVC. Using the *cis*-eQTL criteria, a negative association was found for sICAM (beta: −0.1, 95%CI: −0.16 to −0.04, *p* = 1.0 × 10^−3^) (FDR<5%) (Figures 1, 2, 3, and 4).

#### Peak expiratory flow rate

Using the *cis*-pQTL and *cis*-eQTL instrument definition criteria, no FDR significant association was found (Figures 1, 2, 3, and 4).

All significant (FDR<5%) and nominally significant (*p* < 0.05) associations for all outcomes are shown in Tables S2 and S3.

Analysis showed similar results in weighted-median, MR-Egger and MR-PRESSO for all FDR significant associations, suggesting little evidence for directional pleiotropy driving the results (Tables S2 and S3).

### Review of databases for medical drugs

For the 8 cytokines (MCSF, IL-18, IL-1ra, sICAM, MCP3, PDGFbb, sVCAM, TNF-a) that showed evidence of a causal association with COVID-19 outcomes, asthma, COPD or pulmonary function indices in the main (*cis*-pQTL) MR analyses, records on clinical drug development programs were identified for IL-18, IL-1ra, MCSF, sICAM, sVCAM, PDGFbb, and TNF-a, and some of the drugs have already been marketed. Some of the indications and associated conditions for the investigational and approved drugs are skin disorders, multiple sclerosis, and acute lymphoblastic leukemia (Table S4).

### Colocalization

Overall, the findings from colocalization analyses supported most of the FDR significant associations. Poor evidence for shared causal variants was found for MCSF with risk of hospitalization due to COVID-19, CTACK with risk of COPD, IL-1ra with FVC, PDGFbb with FEV_1_, and sICAM with FEV_1_/FVC, which might indicate genetic confounding in those associations or be a result of limited power (weak signals) (Tables S5 and S6).

### Secondary traits associated with selected instruments and sensitivity analyses

Removal of the variants associated with anthropometric indices and height in the PhenoScanner database^18^ (Table S7) from the cytokine genetic instruments that showed evidence of associations with any of the examined outcomes, did not alter the results; the only exceptions were the associations between MCSF and FVC, which did not remain significant, and between TNF-a and atopic asthma, for which the only instrument was associated with height, weight and hip circumference (Table S8). In sensitivity analyses adjusting for the slight correlation among variants (r^2^ < 0.1), no differences were observed (Table S9). Additionally, iterative leave-one-out analysis showed that no association was driven by influential SNPs (Figures S1–S15). The secondary analysis using the eQTL cytokines in lung tissue produced qualitatively similar results to the main eQTL analysis, but associations were nominally significant only for IL-18 in relation to FEV1, FVC, and COPD, and IL-16 in relation to atopic asthma (Table S10).

### Observational analysis in the UK biobank

The positive association of IL-18 on COPD (OR: 1.31, 95%CI: 1.21 to 1.42, *p* < 0.001), and the inverse associations of IL-18 on FEV_1_ (−0.061, 95%CI: −0.071 to −0.051, *p* < 0.001) and FVC (−0.079, 95%CI: −0.093 to −0.065, *p* < 0.001), were replicated in an observational analysis in the UK Biobank. The inverse association of MCSF on FVC (−0.129, 95%CI: −0.152 to −0.105, *p* < 0.001) was also replicated. The rest of the associations were not replicated either because they were non-significant, or because they were in the opposite direction compared to the MR analysis (Table S11).

## Discussion

This hypothesis-free exploratory analysis provides genetic evidence implicating several inflammatory cytokines in respiratory disease risk. The combined consideration of genetic variants that strongly associate with cytokine protein and gene expression maximizes the potential to identify genetic proxies of cytokine effects, with the exploration of effects on measures of respiratory function, respiratory disease, and COVID-19 serving to identify distinct roles across respiratory disease phenotypes.

Regarding COVID-19 risk, we found evidence supporting the protective effects of MCSF, sICAM, and sVCAM. In contrast, a previous conventional epidemiological association study identified associations of sICAM and sVCAM with worse outcomes in COVID-19.^19^ The discrepancy with our current findings may be attributable to the potential for the previously described epidemiological associations to be affected by confounding factors and reverse causation, while MR is less vulnerable to such sources of spurious associations. However, an alternative and maybe more plausible explanation is that genetically predicted high levels of these cytokines indicate a genetic profile for a strong immune response that may protect from severe disease; but high directly measured cytokine levels in an observational setting indicate the severity of the COVID-19 or the percentage of the lung being involved in the infection. The evidence supporting the protective effect of MCSF is consistent with its biological role in the differentiation of macrophages. A recent MR study found very similar results for COVID-19 outcomes with our work, using a different GWAS for cytokines.^20^

Considering lung function, IL-1ra is known to modulate IL1-driven inflammation, thus improving FEV_1_ as suggested by our current genetic analyses. Perhaps a surprising finding in our analyses was the evidence for higher circulating MCP3 levels increasing FEV_1_ and FVC, as this chemokine is implicated in the recruitment of inflammatory cells including eosinophils to the lung,^21^ which would be expected to worsen respiratory function. This discrepancy may be reconciled to its effects being locally exerted. Thus, as its circulating levels are increased, this may paradoxically have favorable effects on reducing eosinophil recruitment in the lung environment. IL-18 is an inflammatory cytokine that has previously been implicated in the pathophysiology of COPD.^22^^,^^23^^,^^24^ The current genetic and observational analyses support this harmful effect and further identify similar potential effects on reducing FEV_1_ and FVC, thus additionally highlighting potential disease mechanisms.

The methodological approach taken in these genetic analyses also offers broader mechanistic insights. The complementary consideration of circulating cytokine levels as well as cross-tissue gene expression provides evidence for the role of sICAM and sVCAM in COVID-19 and IL-1ra in FEV_1_. Further, by incorporating distinct phenotypic traits reflecting various aspects of respiratory function, we were able to unravel specific and widespread effects of cytokines on each of these. Demonstrating this, the consistent evidence for sICAM across the considered COVID-19 phenotypes supports its role in this disease. Furthermore, the opposite effects of sICAM on the risks of COVID-19 and atopic asthma (inverse and positive, respectively), reinforce the negative association between these diseases found in a previous MR study.^25^

Finally, our triangulation of genetic evidence with existing observational associations provides complementary insight through methodological approaches that vary in their underlying modeling assumptions, thus strengthening the overall evidence for clinical pursuit.^26^ The presence of existing clinical drug development programs for IL-18, IL-1ra, MCSF, sICAM, sVCAM, PDGFbb, and TNF-a may support the prioritization of these targets for exploration in the remit of chronic respiratory disease.

### Limitations of the study

Our work also has limitations. We examined the associations of 35 inflammatory cytokines with respiratory diseases and pulmonary function indices; however, these constitute a small portion of the total number of inflammatory cytokines, and future studies could elucidate associations with further inflammatory cytokines, as their genetic determinants are better researched. The genetic variants used to proxy the perturbation of cytokine effects reflect small, lifelong changes, which may not mimic the effect of a discrete pharmacological intervention in adult life. The availability of genetic variants to serve as instruments for some of the considered cytokines was limited, thus further robust MR analyses could not be pursued. Furthermore, some of the colocalization analyses were likely underpowered and were thus not able to reliably provide any assurance against confounding by LD. We used data for European ancestry individuals, hence our results are not generalizable to other ethnicities. Additionally, previous relevant epidemiological data are relatively scarce; therefore, the validation of our results with further studies is needed. Finally, there remains the prospect that any identified associations of genetic variants proxying cytokine levels may be related to pleiotropic effects unrelated to cytokine effects, thus violating the requisite MR modeling assumptions. While the use of variants at the genes coding for the respective cytokines that are related to their circulating protein levels or gene expression provides some assurances against this, it does remain a possibility. The observational analyses can capture different components of the associations (largely reflecting protein concentrations cross-sectionally) compared to MR (that proxy the effects of the genetically predicted concentrations), and the observational analyses are prone to biases such as confounding or reverse causation that may explain the observed differences in findings from the two approaches.

In conclusion, we have leveraged large-scale genetic association data related to cytokine protein and gene expression to gain novel mechanistic insight into the role of these inflammatory mediators in chronic lung diseases, lung function, and COVID-19. Our findings can be used to prioritize therapeutic targets for further study across respiratory disease areas, potentially increasing the probability of successful drug development, while also reducing the associated time and cost.

## Resource availability

### Lead contact

Further information and requests for resources should be directed to and will be fulfilled by the lead contact, Konstantinos K. Tsilidis (k.tsilidis@imperial.ac.uk).

### Materials availability

The study did not generate any new materials.

### Data and code availability

• All data used in this work are presented in the online data supplement and are available in the original publications.
• The code used for this paper is available in Supplementary Text.
• Any additional information required to reanalyze the data reported in this paper is available from the lead contact upon request.
• The paper has been written in accordance with the Strengthening the Reporting of Observational Studies in Epidemiology using Mendelian Randomization (STROBE-MR) guidelines (Supplementary Text).^27^

## Acknowledgments

This study is co-financed by the 10.13039/501100008530European Regional Development Fund of the European Union and Greek national funds through the Operational Program “Competitiveness, Entrepreneurship and Innovation (EPAnEK), NSRF 2014-2020 (Project code 10.13039/501100013296MIS: OΠΣ 5047228).” The Young Finns Study has been financially supported by the 10.13039/501100002341Academy of Finland: grants 322098, 286284, 134309 (Eye), 126925, 121584, 124282, 255381, 256474, 283115, 319060, 320297, 314389, 338395, 330809, and 104821, 129378 (Salve), 117797 (Gendi), and 141071 (Skidi); the 10.13039/501100002327Social Insurance Institution of Finland; Competitive State Research Financing of the Expert Responsibility area of Kuopio, Tampere and Turku University Hospitals (grant X51001); 10.13039/501100004037Juho Vainio Foundation; 10.13039/501100008484Paavo Nurmi Foundation; 10.13039/501100005633Finnish Foundation for Cardiovascular Research; 10.13039/501100003125Finnish Cultural Foundation; The Sigrid Juselius Foundation; 10.13039/501100006706Tampere Tuberculosis Foundation; 10.13039/501100004756Emil Aaltonen Foundation; 10.13039/100010114Yrjö Jahnsson Foundation; 10.13039/501100004325Signe and Ane Gyllenberg Foundation; Diabetes Research Foundation of Finnish Diabetes Association; EU Horizon 2020 (grant 755320 for TAXINOMISIS and grant 848146 for To Aition); 10.13039/501100000781European Research Council (grant 742927 for MULTIEPIGEN project); Tampere University Hospital Supporting Foundation, Finnish Society of Clinical Chemistry and the Cancer Foundation Finland. NFBC1966 31-year follow up that included cytokine measurements received financial support from 10.13039/501100006196University of Oulu Grant no. 65354, Oulu University Hospital Grant no. 2/97, 8/97, Ministry of Health and Social Affairs Grant no. 23/251/97, 160/97, 190/97, National Institute for Health and Welfare, Helsinki Grant no. 54121, Regional Institute of Occupational Health, Oulu, Finland Grant no. 50621, 54231. The data generation, curation and manpower were also supported by the following EU H2020 grants: DynaHEALTH (grant no 633595), LifeCycle (733206), LongITools (873749), EarlyCause (848158), EDCMET (825762) and the 10.13039/501100000265Medical Research Council, UK: grant number MRC/BBSRC MR/S03658X/1 (JPI HDHL H2020).

## Author contributions

K.K.T., M-R.J., and A.D. conceived and designed the study. M.O.R., E.B., and A-G.A. performed the statistical analyses. M.O.R. and E.B. have directly accessed and verified the underlying data. All authors had full access to all the data in the study and interpreted the results. M.O.R. and D.G. drafted the article. All authors critically revised the article for intellectual content. All authors read and approved the final article. All authors accept responsibility to submit for publication.

## Declaration of interests

The authors declare the following competing interests: Salomaa Veikko was funded by the Finnish Foundation for Cardiovascular Research and by the Juho Vainio Foundation. Salomaa Veikko has received an honorarium from Sanofi for consulting and has ongoing research collaboration with Bayer Ltd. (All outside the present study). These companies had no role in study design, data collection and analysis, decision to publish, or preparation of the article. Sirpa Jalkanen, Marko Salmi: Financial support for cytokine analyses has come from the Finnish Academy. Abril Izquierdo and Martin Tobin were supported for the present article by Wellcome Trust grants (WT202849/Z/16/Z, WT225221/Z/22/Z) and the National Institute of Health and Care Research (NIHR) Leicester Biomedical Research Center. Martin Tobin has also received an NIHR Senior Investigator Award and had a funded research collaboration with Orion Pharma (the latter outside the scope of this work). Markku Timonen has received payment for one lecture for Otsuka Pharmaceutical and payment for two lectures for H. Lundbeck A/S. Marjo-Ritta Jarvelin is partly funded by the Medical Research Council (MR/S019669/1). James Yarmolinsky is supported by a Cancer Research UK Population Research Postdoctoral Fellowship (C68933/A28534). The remaining authors have no competing interests to declare.

## STAR★Methods

### Key resources table

**Table:** 

REAGENT or RESOURCE	SOURCE	IDENTIFIER
**Deposited data**		

Cytokines	Sun et al.^15^	https://pubmed.ncbi.nlm.nih.gov/29875488/
Cytokines	Karhunen et al.^16^	https://www.ncbi.nlm.nih.gov/pmc/articles/PMC9978757/
Cytokines	Folkersen et al.^14^	https://pubmed.ncbi.nlm.nih.gov/33067605/
COVID-19 outcomes	The COVID-19 host genetics initiative	https://www.covid19hg.org/
Asthma	Zhu et al.^28^	https://pubmed.ncbi.nlm.nih.gov/31669095/
COPD	Sakornsakolpat et al.^29^	https://pubmed.ncbi.nlm.nih.gov/30804561/
Pulmonary function indices	Shrine et al.^30^	https://www.ncbi.nlm.nih.gov/pmc/articles/PMC6397078/

**Software and algorithms**		

R	version 4.0.2.	https://www.r-project.org
MendelianRandomization	version 0.6.0.	https://cran.r-project.org/
coloc	version 5.2.3.	https://cran.r-project.org/

### Experimental model and study participant details

#### Cytokine instrument selection

We used summary genetic association estimates from a GWAS meta-analysis of circulating levels of 47 inflammatory cytokines from the Northern Finland Birth Cohort 1966 (NFBC1966),^17^ the Cardiovascular Risk in Young Finns (YFS) study, FINRISK 1997 and 2002, the INTERVAL study and the SCALLOP consortium,^13^^,^^14^^,^^15^^,^^16^ including up to 31,112 individuals (ranging from 3,301 to 31,112) (Table S12; Figure 5). We present the summary data of this GWAS in Table S13 and we provide more details of the GWAS meta-analysis rationale and analytical plan in a recent publication.^31^

We used *cis* instrument definitions, selecting genetic variants located in or close to the coding gene, thus we minimized the probability of horizontal pleiotropy (when variants influence the outcomes of interest through pathways other than the cytokines of interest) and we provided association estimates less likely to be affected by this bias.^10^^,^^16^^,^^32^ In the main instrument definition, we selected genetic variants with a minor allele frequency (MAF) > 0.05, within ±500 kilobases (kb) of the corresponding cytokine gene locus, associated with the circulating cytokine concentrations at *p* < 1 × 10^−4^; we termed these genetic variants ‘*cis*-protein quantitative trait locus’ (*cis*-pQTL) (Table S13). To validate the main analysis and potentially capture additional associations we used a ‘cis-expression quantitative trait locus’ (cis-eQTL) definition, selecting genetic variants within ±500kb of the corresponding cytokine gene locus, associated with both its gene expression aggregated across tissues at *p* < 1×10^-4^ (using data from Genotype-Tissue Expression (GTEx) database),^33^ and its circulating cytokine concentrations at *p* < 0.05.^16^ In a secondary analysis and to validate the results of the eQTL analysis, we repeated the analysis using a slightly altered eQTL definition, selecting genetic variants using the same criteria but expressed only in lung tissue.

To avoid the potential loss of causal variants via clumping, we used a lenient clumping r^2^ threshold of 0.1, and we evaluated the robustness of the significant associations in sensitivity analysis, accounting for the LD structure.^34^ More details for the methods used are provided in Supplementary Text.

#### Outcome data

##### COVID-19

We used summary genetic association estimates from the COVID-19 host genetics initiative (https://www.covid19hg.org/results/) for the risks of four COVID-19 outcomes, namely (i) Any COVID-19, (ii) COVID-19 hospitalization, (iii) Severe COVID-19, (iv) Case only COVID-19 hospitalization, using GWAS data for European ancestry individuals. More details on the COVID-19 outcomes are provided in Supplementary Text.

##### Asthma, COPD and pulmonary function indices

Genetic association estimates for asthma were obtained from a UK Biobank GWAS, including 24,600 cases and 432,368 controls of European ancestry for atopic asthma, and 29,605 cases and 319,321 controls for non-atopic asthma.^28^ Estimates for COPD were selected from a GWAS in 35,735 cases and 222,076 controls, using data from 25 studies (including UK Biobank and studies from the International COPD Genetics Consortium).^29^ Genetic association estimates for indices of pulmonary function, namely FEV_1_, FVC, FEV_1_/FVC and PEF, were obtained from a GWAS in individuals of European ancestry (for FEV_1_, FVC and FEV_1_/FVC 321,047 samples were available from UK Biobank and 79,055 from the SpiroMeta Consortium; for PEF up to 24,218 samples from SpiroMeta).^30^

#### Ethics approval and consent to participate

All studies contributing data to these analyses had the relevant institutional review board approval from each country, in accordance with the Declaration of Helsinki, and all participants provided informed consent.

### Method details

#### Mendelian randomization analyses

We used the inverse-variance weighted (IVW) MR method^35^ in the case of multiple genetic instruments or the ratio of coefficients method in the case of a single instrument, to investigate the associations of genetically proxied circulating cytokine concentrations in relation to the outcomes. To account for multiple comparisons we estimated the FDR adjusted p values (q-values), as proposed by Benjamini and Hochberg, and considered q-values of less than 5% as statistically significant.^36^

We assessed the MR assumptions, namely (i) relevance (genetic variants strongly associated with the cytokine concentrations), (ii) exchangeability (no common causes of the instrument-outcome association), and (iii) exclusion restriction (genetic variants only influence outcomes via the inflammatory cytokines) using several approaches. To examine the first assumption, we calculated the F-statistic and proportion of variance explained (r^2^) that measure the strength of each genetic variant, and excluded weak instruments (i.e. with F-statistic<10) from the analysis (Table S13).^37^^,^^38^ To assess the second and third MR assumptions, we searched the PhenoScanner database^18^ to identify secondary traits (non-inflammatory, potentially pleiotropic traits) associated with the genetic instruments in previous GWAS. The most common violation of the third MR assumption is horizontal pleiotropy that occurs when a variant influences the outcome through phenotypes other than the exposure being instrumented. We have, in part, adjusted for horizontal pleiotropy “by design” with our cis genetic instrument definition. We further used additional MR analyses that are more robust to violations of the exclusion restriction assumption, namely the weighted-median,^39^ MR-Egger,^40^ and MR-PRESSO method,^41^ with the caveat that these methods operate best in a polygenic MR analysis framework.

#### Colocalization and leave-one-out analysis

We performed colocalization analyses to investigate whether the observed MR associations may have resulted from confounding by LD (i.e. exposure and outcome not having shared causal variants).^42^ This method is described in detail in Supplementary Text. To examine whether associations were driven by a single influential SNP in the associations that survived colocalization analysis we performed iterative leave-one-out analysis, iteratively removing one SNP at a time from instruments.

#### Review of publicly available databases for medication

For the cytokines that emerged via the MR analyses as potentially relevant to our outcomes, we searched the DrugBank database (https://go.drugbank.com/) to identify drugs targeting them, and for the identified drugs, we searched the widely used clinical trial protocol repository of clinicaltrials.gov (https://clinicaltrials.gov/).

#### Observational analysis in the UK biobank

The associations that emerged in the MR analyses were explored in the UK Biobank,^43^ which is an ongoing prospective cohort study, which enrolled 502,412 participants aged 40 to 69 years in the UK between 2006 and 2010. Blood plasma samples were randomly selected from 54,306 UK Biobank participants, capturing 1,463 unique proteins.^44^ The association of circulating plasma protein concentrations and pertinent outcomes was evaluated using regression models. Further details on the analysis are presented in Supplementary Text.

### Quantification and statistical analysis

All analyses were performed using R, version 4.0.2.^45^ Two-sample MR analyses were conducted using the MendelianRandomization package (version 0.6.0.). Colocalization analyses were conducted using the coloc package (version 5.2.3). Figures were constructed using the forestplot (version 3.1.3.) and ggplot2 packages (version 3.4.3) (available from the R CRAN repository (https://cran.r-project.org/)).

MR estimates reflect the change in outcome per one standard deviation (SD) higher concentration in the natural scale of each cytokine.

## Supplemental information

Document S1. Figures S1–S15 and Data S1–S3

Table S1. List of cytokines, related to STAR Methods

Table S2. Summary of *cis*-pQTL results, related to Figures 1 and 3

Table S3. Summary of *cis*-eQTL results, related to Figures 2 and 4

Table S4. Druggable targets, related to STAR Methods

Table S5. Colocalization analysis of significant *cis*-pQTLs and *cis*-eQTLs, related to STAR Methods

Table S6. Colocalization with SuSie, related to STAR Methods

Table S7. Associated traits from Phenoscanner, related to STAR Methods

Table S8. Sensitivity analysis excluding variants associated with somatometric indices or height in Phenoscanner, related to STAR Methods

Table S9. Sensitivity analysis adjusting for the genetic correlation among variants, related to STAR Methods

Table S10. Sensitivity analysis for eQTL cytokines in lung tissue, related to STAR Methods

Table S11. Summary of the results from observational analysis in the UK Biobank, related to STAR Methods

Table S12. Source of GX, related to STAR Methods

Table S13. GX instruments, related to STAR Methods
