# Supplementary material for: Association of inflammatory cytokines with lung function, chronic lung diseases, and COVID-19
Source: iScience. 2024 Aug 9;27(10):110704. doi: 10.1016/j.isci.2024.110704 (PMC11417323; doi:10.1016/j.isci.2024.110704)
Supplement: Document S1. Figures S1–S15 and Data S1–S3 [file mmc1.pdf]

iScience, Volume *27*

Supplemental information

Association of inflammatory cytokines with lung function, chronic lung diseases, and COVID-19

Marina O. Rontogianni, Dipender Gill, Emmanouil Bouras, Alexandros-Georgios Asimakopoulos, Ioanna Tzoulaki, Ville Karhunen, Terho Lehtimäki, Olli Raitakari, Matthias Wielscher, Veikko Salomaa, Sirpa Jalkanen, Marko Salmi, Markku Timonen, James Yarmolinsky, Jing Chen, Martin D. Tobin, Abril G. Izquierdo, Karl-Heinz Herzig, Anne E. Ioannides, Marjo-Riitta Jarvelin, Abbas Dehghan, and Konstantinos K. Tsilidis

Figure S1: Iterative leave-one-out analysis for Mendelian randomisation analysis of genetically-proxied IL1RA and COPD, related to STAR methods

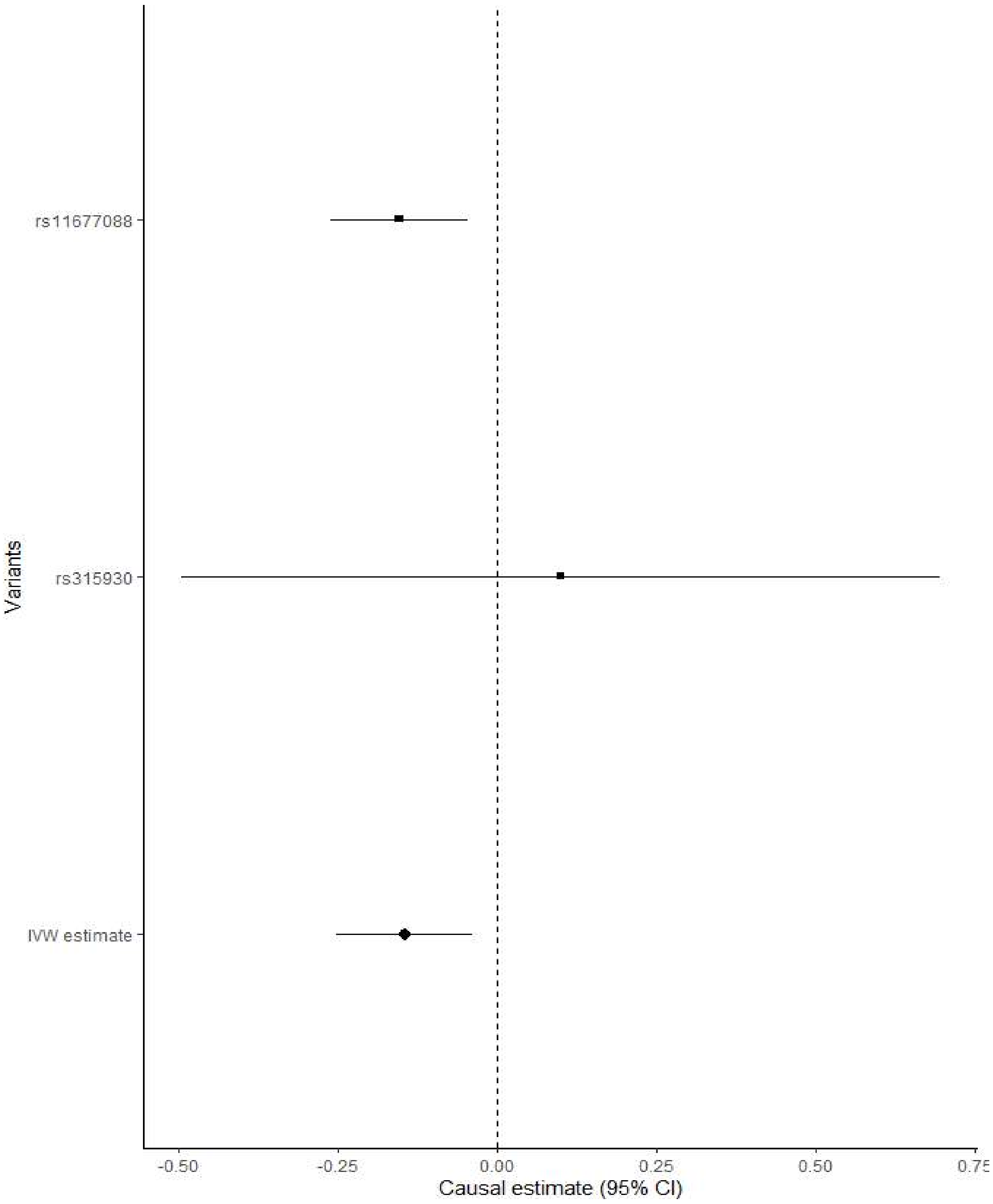

Abbreviations: IVW, Inverse Variance Weighted method; CI, confidence interval; IL1RA, interleukin 1 receptor antagonist; COPD, chronic obstructive pulmonary disease.

Figure S2: Iterative leave-one-out analysis for Mendelian randomisation analysis of genetically-proxied IL1RA and FEV1, related to STAR methods

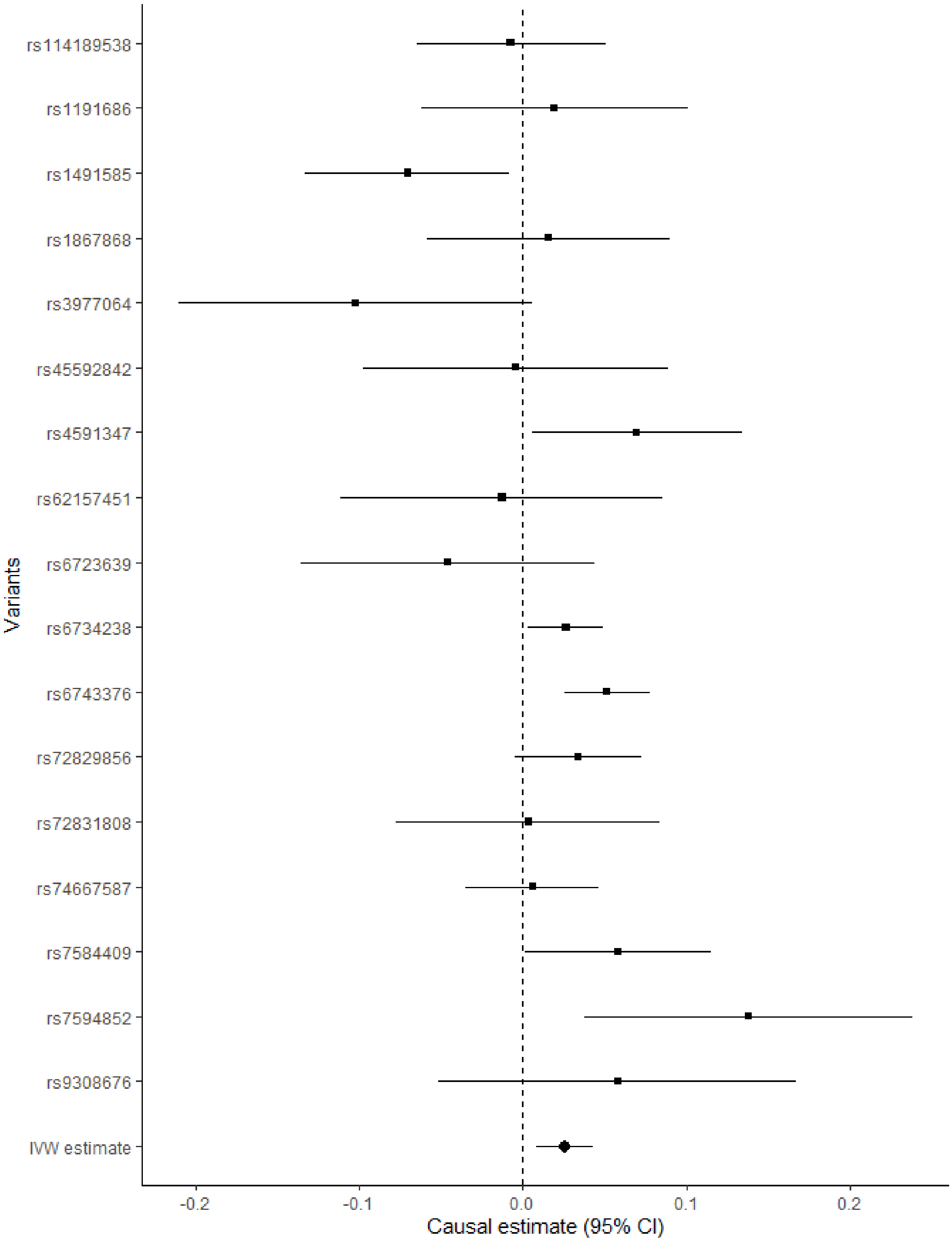

Abbreviations: IVW, Inverse Variance Weighted method; CI, confidence interval; IL1RA, interleukin 1 receptor antagonist; FEV1, forced expiratory volume measured in the first second of exhalation.

Figure S3. Iterative leave-one-out analysis for Mendelian randomisation analysis of genetically-proxied IL18 and COPD, related to STAR methods

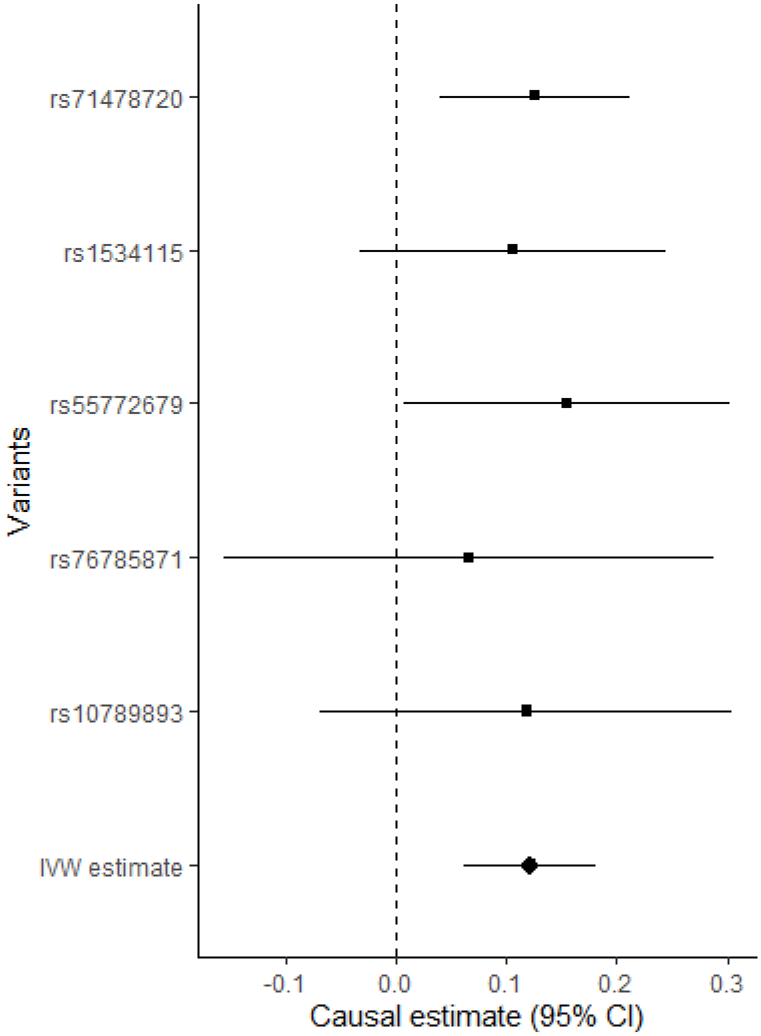

Abbreviations: IVW, Inverse Variance Weighted method; CI, confidence interval; IL 18, interleukin 18; COPD, chronic obstructive pulmonary disease.

Figure S4: Iterative leave-one-out analysis for Mendelian randomisation analysis of genetically-proxied IL18 and FEV1, related to STAR methods

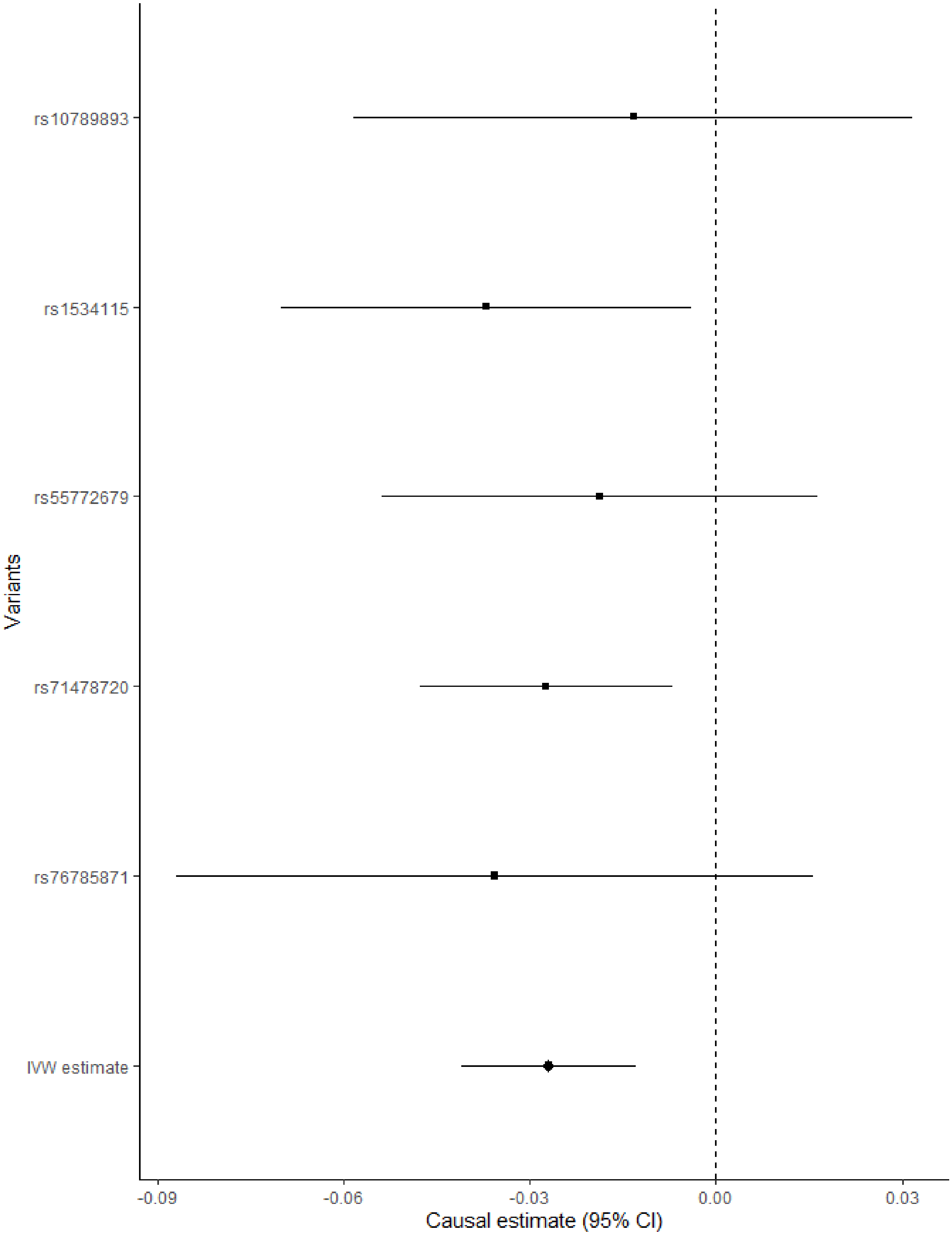

Abbreviations: IVW, Inverse Variance Weighted method; CI, confidence interval; IL 18, interleukin 18; FEV1, forced expiratory volume measured in the first second of exhalation.

Figure S5: Iterative leave-one-out analysis for Mendelian randomisation analysis of genetically-proxied IL 18 and FVC, related to STAR methods

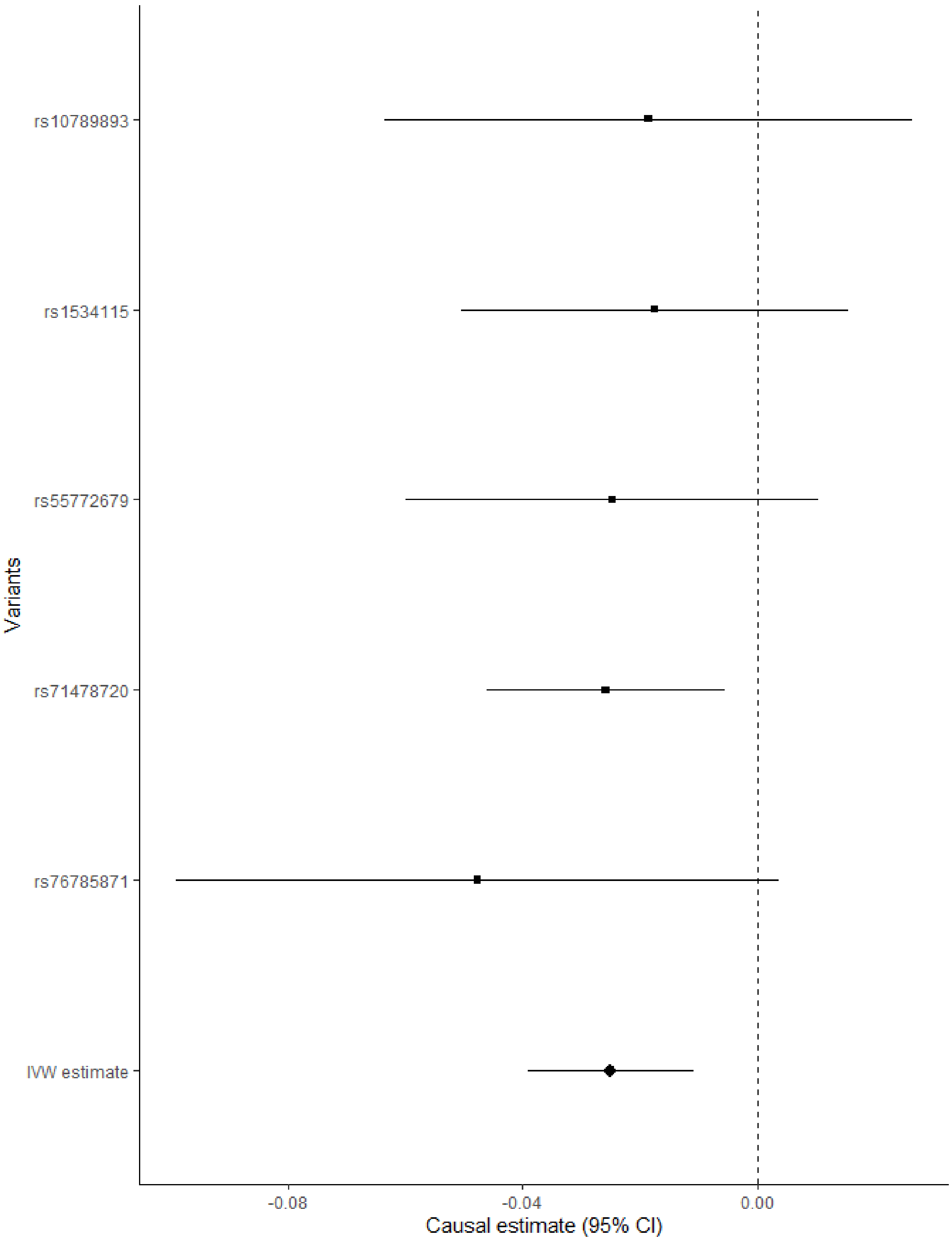

Abbreviations: IVW, Inverse Variance Weighted method; CI, confidence interval; IL 18, interleukin 18; FVC, forced vital

Figure S6. Iterative leave-one-out analysis for Mendelian randomisation analysis of genetically-proxied MCP3 and FEV1, related to STAR methods

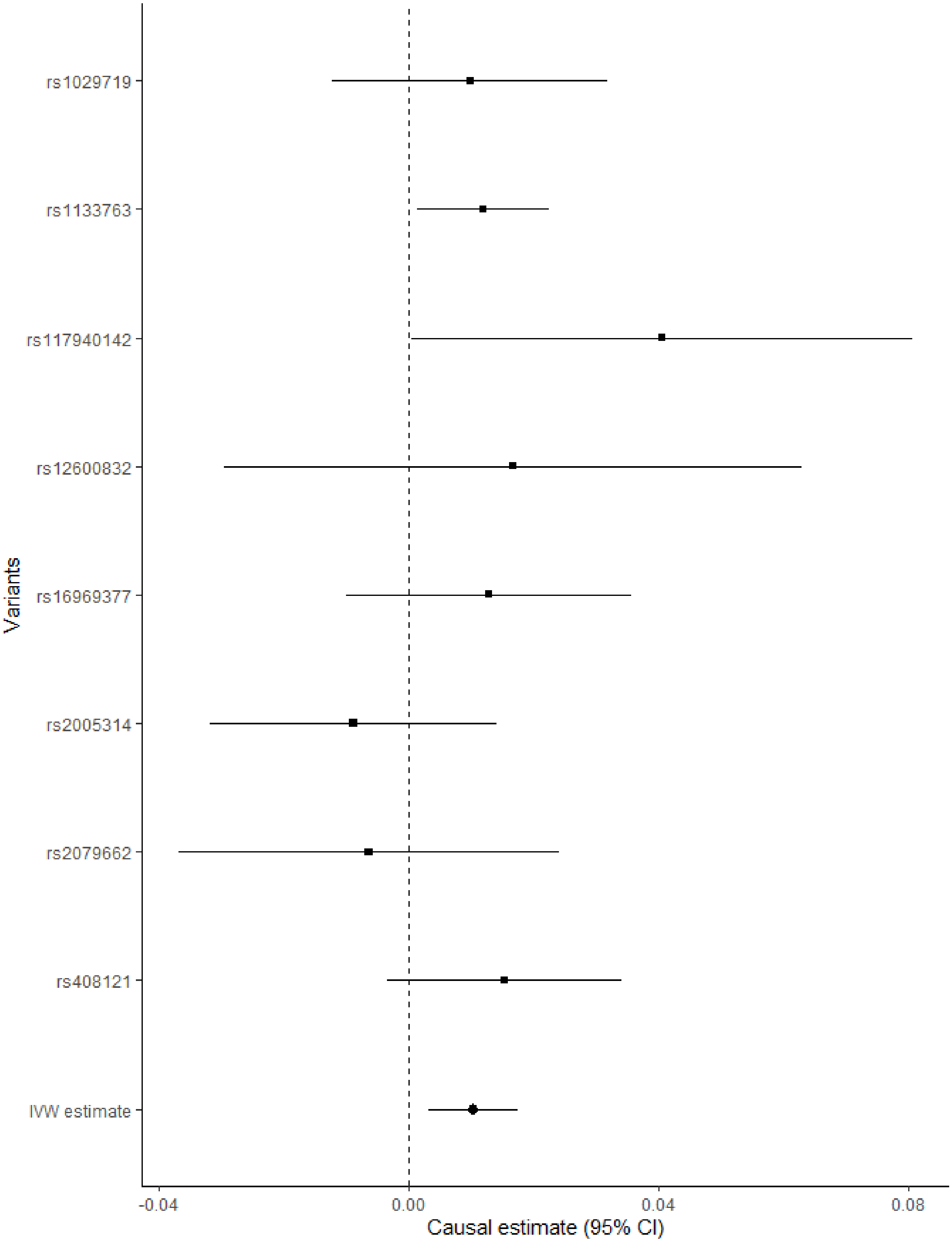

Abbreviations: IVW, Inverse Variance Weighted method; CI, confidence interval; MCP3, monocyte specific chemokine; FEV1, forced expiratory volume measured in the first second of exhalation.

Figure S7: Iterative leave-one-out analysis for Mendelian randomisation analysis of genetically-proxied MCP3 and FVC, related to STAR methods

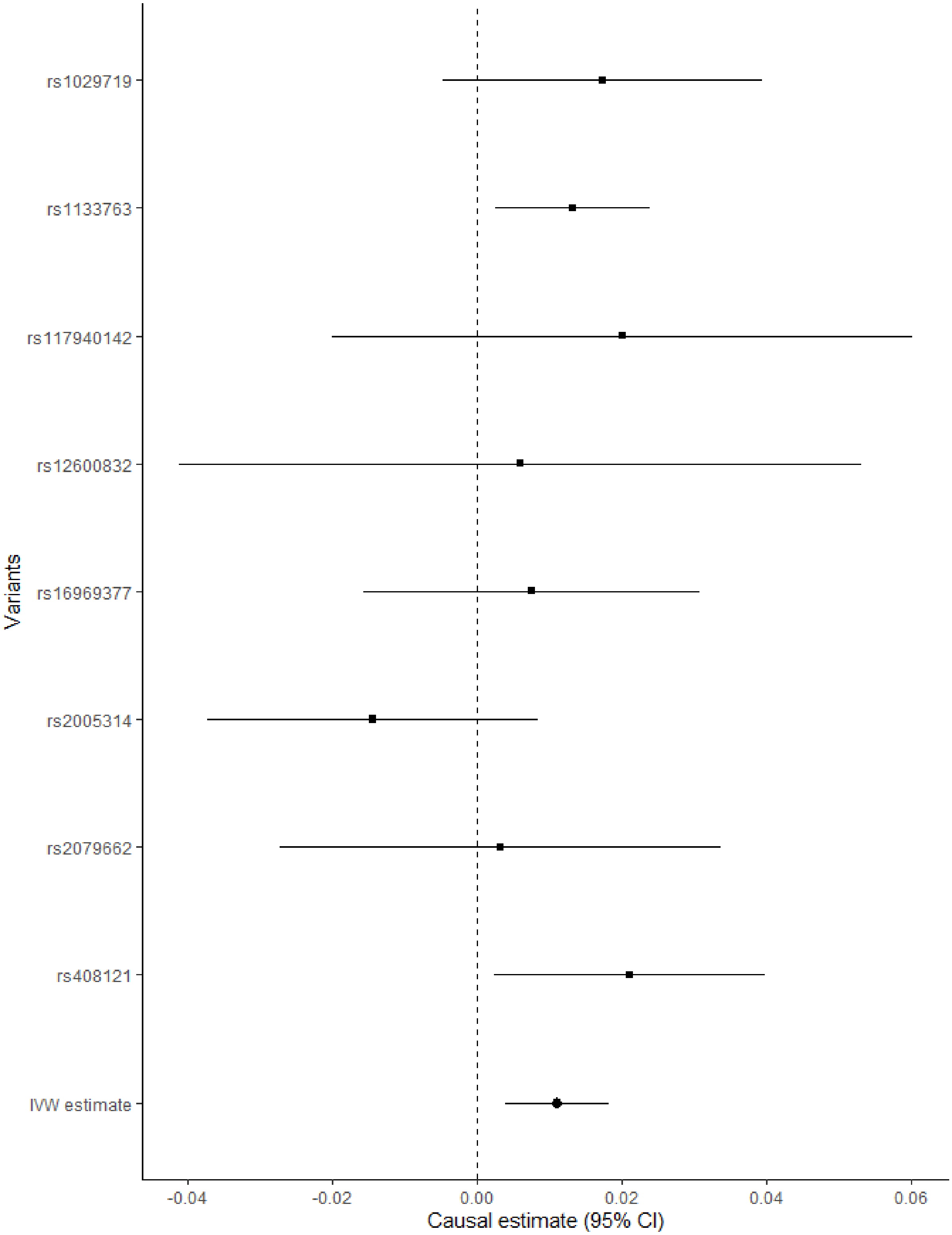

Abbreviations: IVW, Inverse Variance Weighted method; CI, confidence interval; MCP3, monocyte specific chemokine; FVC, forced vital capacity.

Figure S8: Iterative leave-one-out analysis for Mendelian randomisation analysis of genetically-proxied MCSF and COVID-19 (COVID vs. population), related to STAR methods

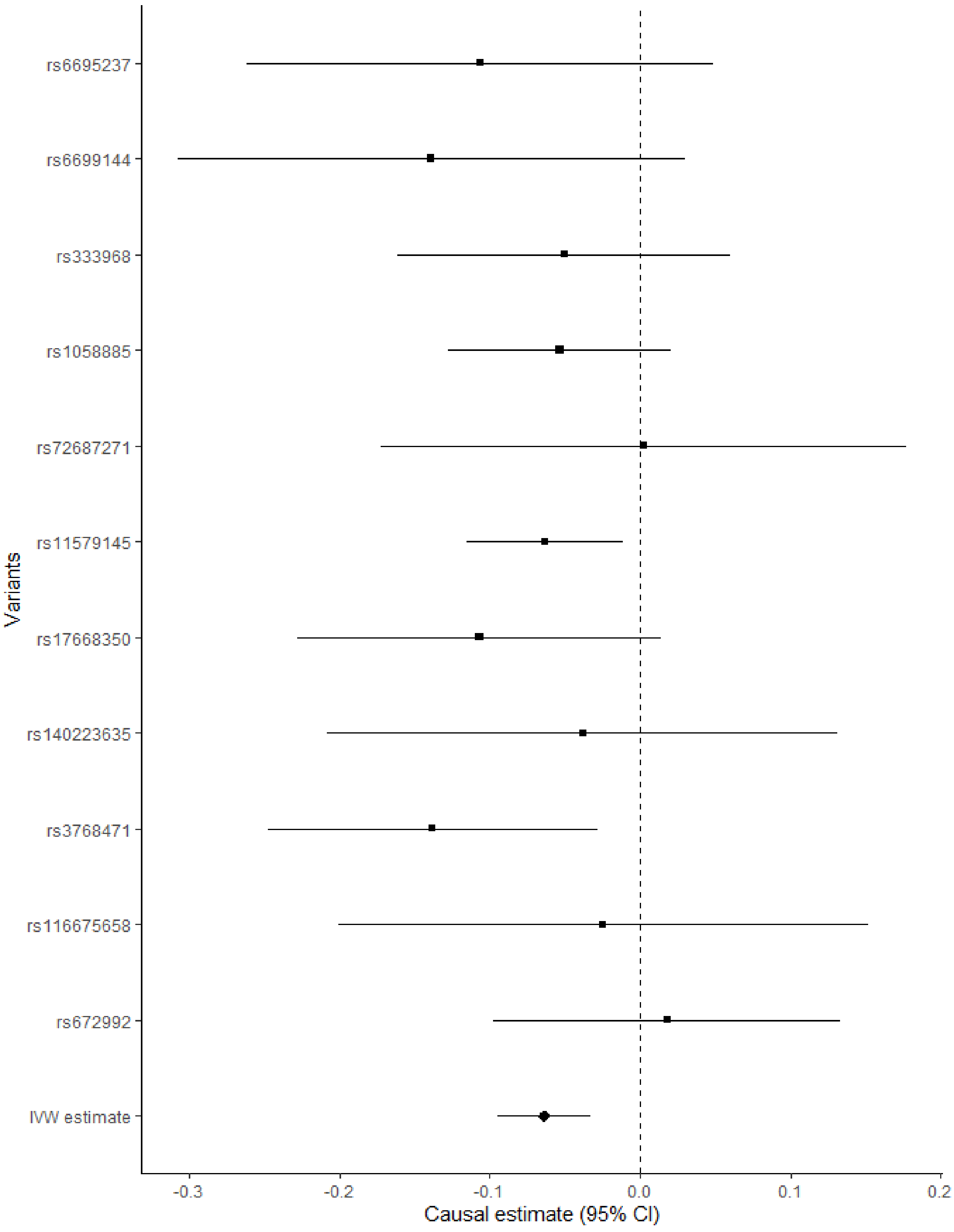

Abbreviations: IVW, Inverse Variance Weighted method; CI, confidence interval; MCSF, macrophage colony-stimulating factor.

Figure S9: Iterative leave-one-out analysis for Mendelian randomisation analysis of genetically-proxied MCSF and FVC, related to STAR methods

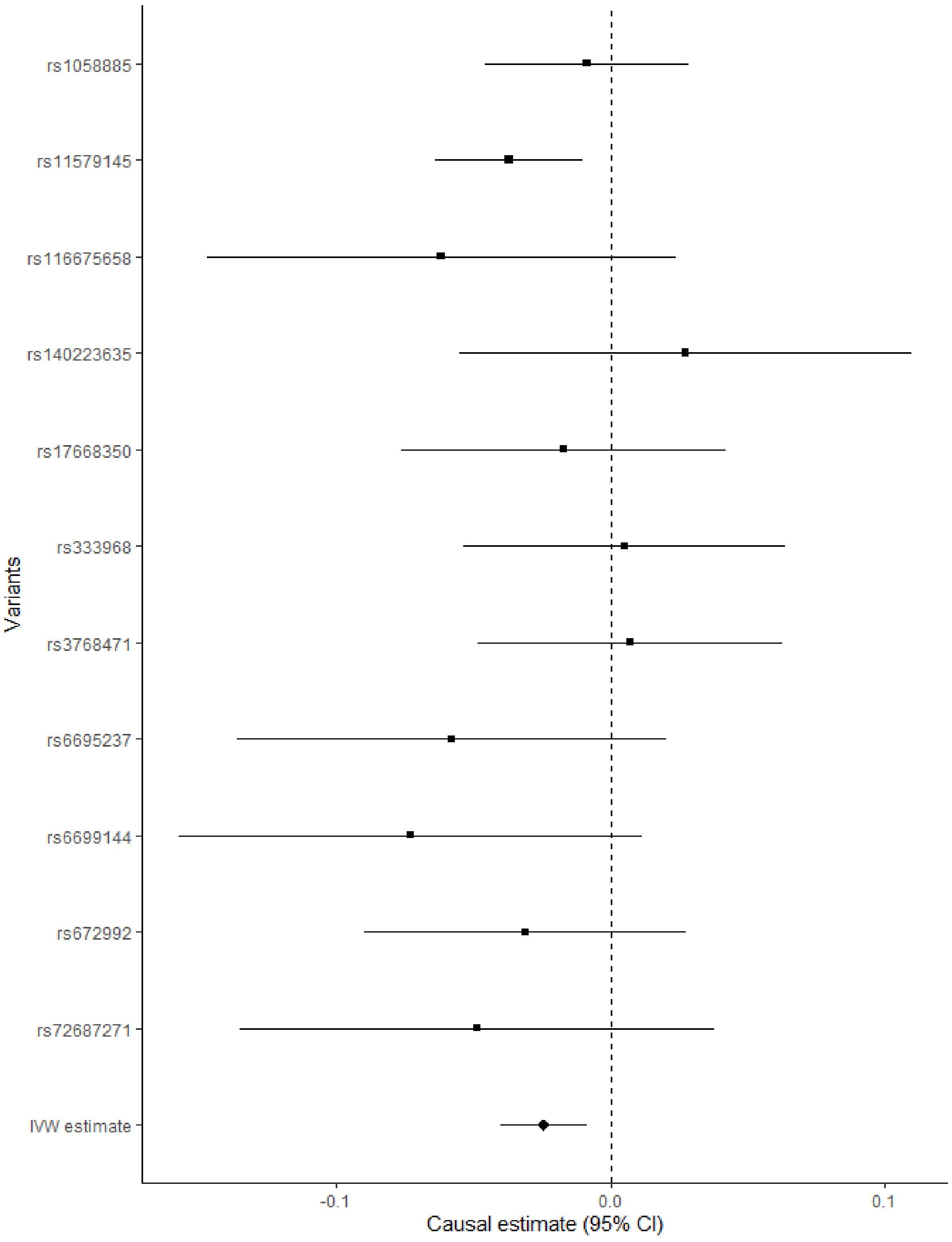

Abbreviations: IVW, Inverse Variance Weighted method; CI, confidence interval; MCSF, macrophage colony-stimulating factor; FVC, forced vital capacity.

Figure S10: Iterative leave-one-out analysis for Mendelian randomisation analysis of genetically-proxied sICAM and COVID- 19 (Severe respiratory COVID vs. population), related to STAR methods

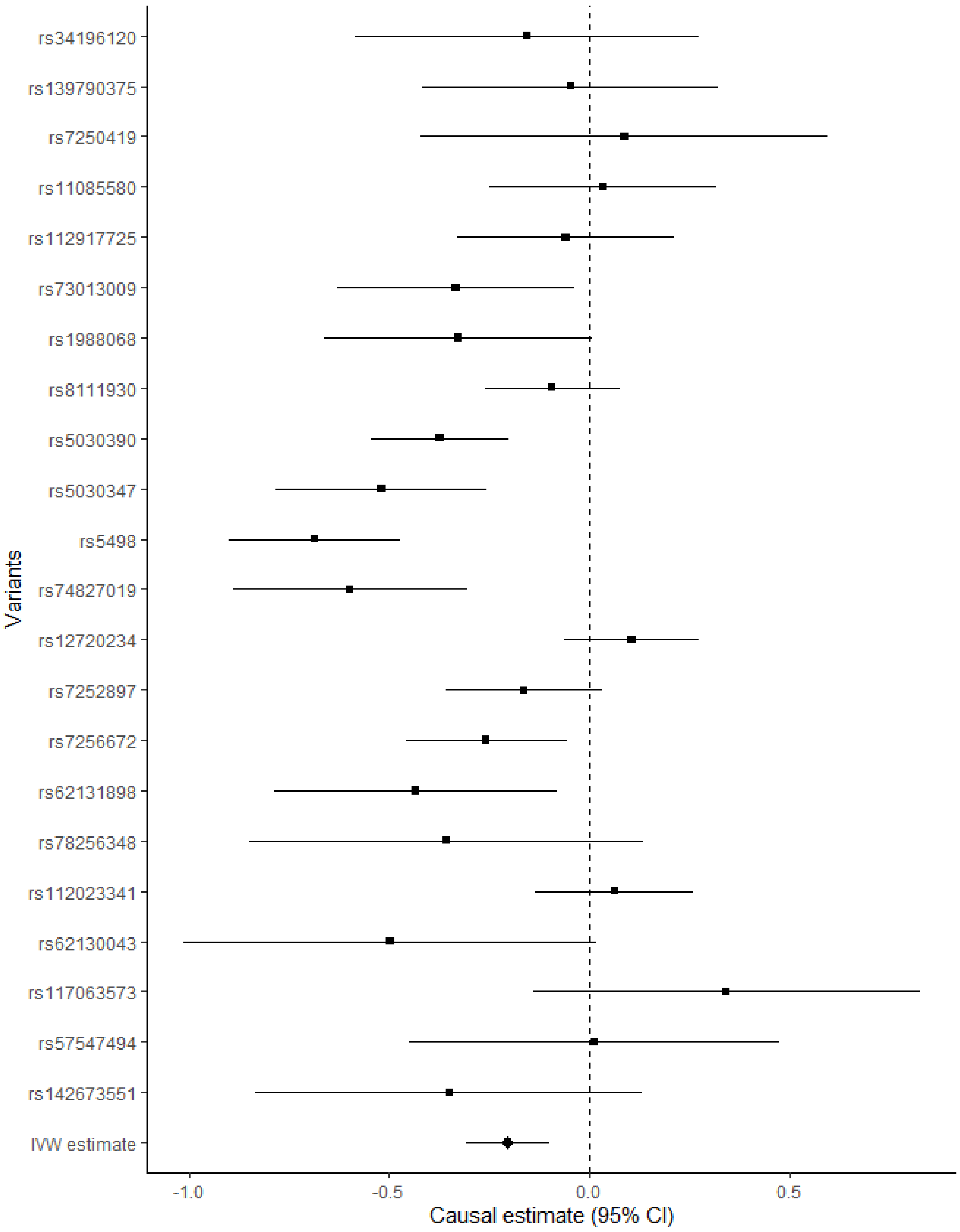

Abbreviations: IVW, Inverse Variance Weighted method; CI, confidence interval; sICAM, soluble intercellular adhesion molecule 1.

Figure S11: Iterative leave-one-out analysis for Mendelian randomisation analysis of genetically-proxied sICAM and atopic asthma, related to STAR methods

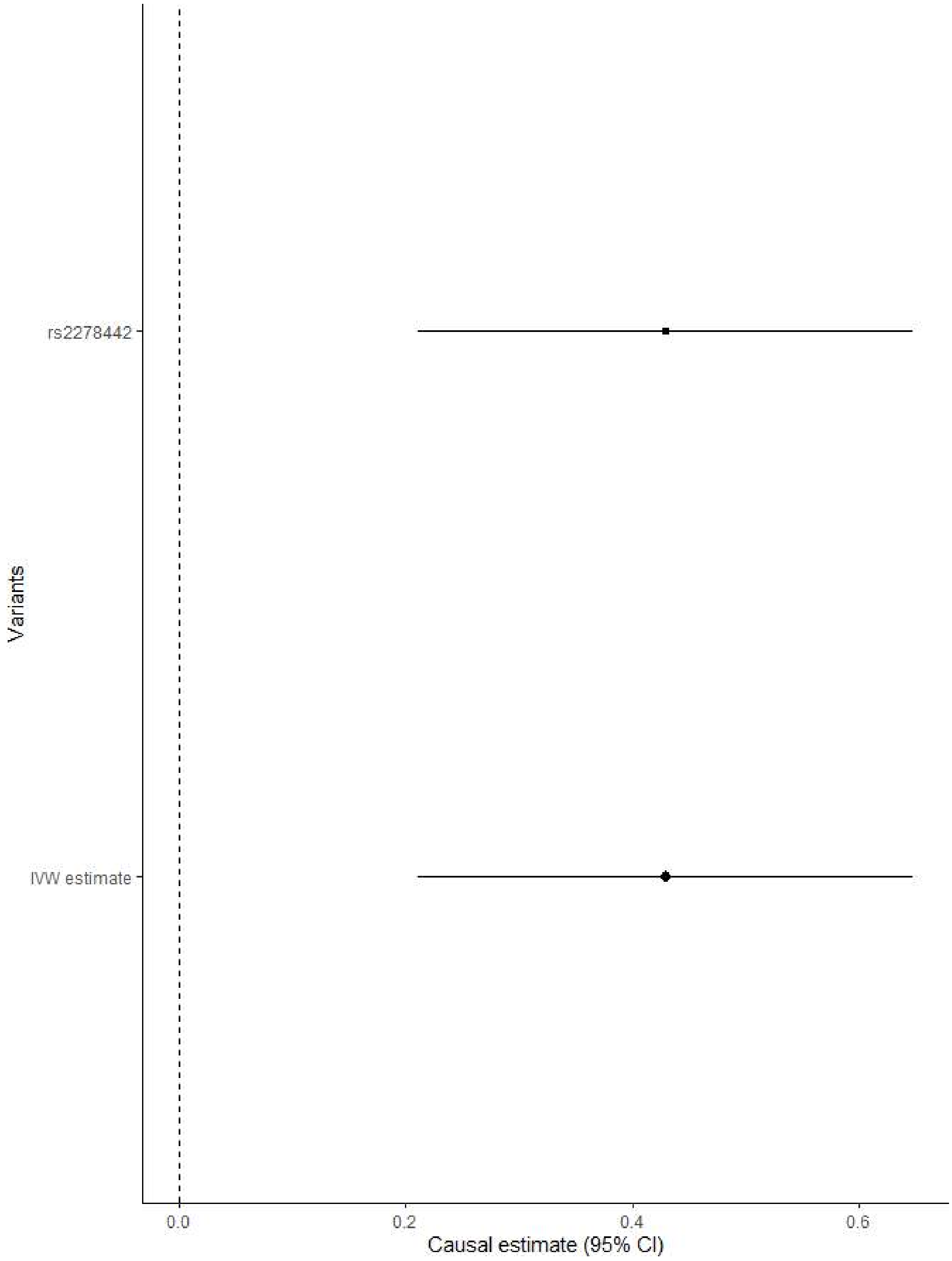

Abbreviations: IVW, Inverse Variance Weighted method; CI, confidence interval; sICAM, soluble intercellular adhesion molecule 1.

Figure S12: Iterative leave-one-out analysis for Mendelian randomisation analysis of genetically-proxied sICAM and COVID- 19 (Hospitalized COVID vs. population), related to STAR methods

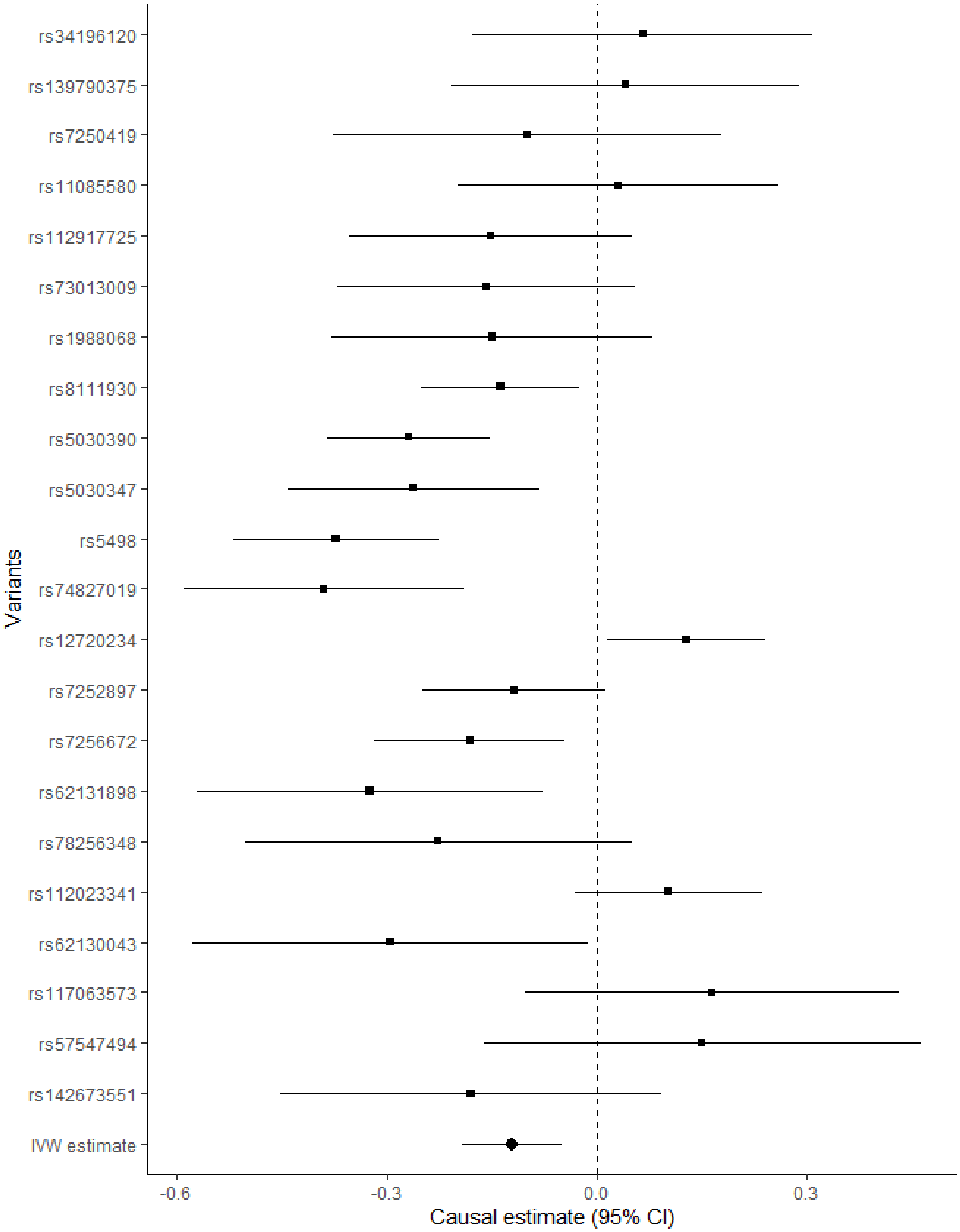

Abbreviations: IVW, Inverse Variance Weighted method; CI, confidence interval; sICAM, soluble intercellular adhesion molecule 1.

Figure S13: Iterative leave-one-out analysis for Mendelian randomisation analysis of genetically-proxied sICAM and COVID- 19 (COVID vs. population), related to STAR methods

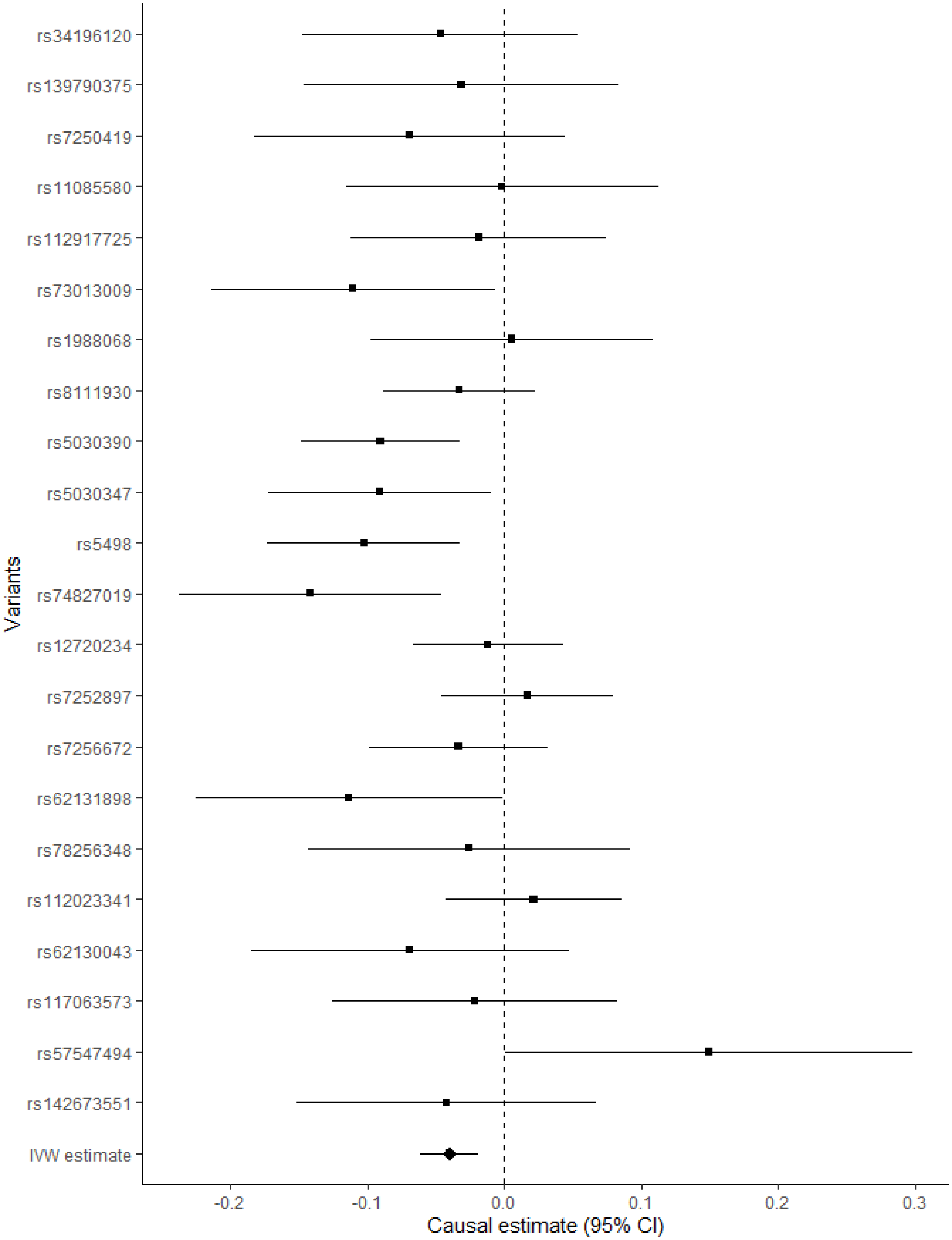

Abbreviations: IVW, Inverse Variance Weighted method; CI, confidence interval; sICAM, soluble intercellular adhesion molecule 1.

Figure S14: Iterative leave-one-out analysis for Mendelian randomisation analysis of genetically-proxied sVCAM and COVID- 19 (Hospitalized COVID vs. population), related to STAR methods

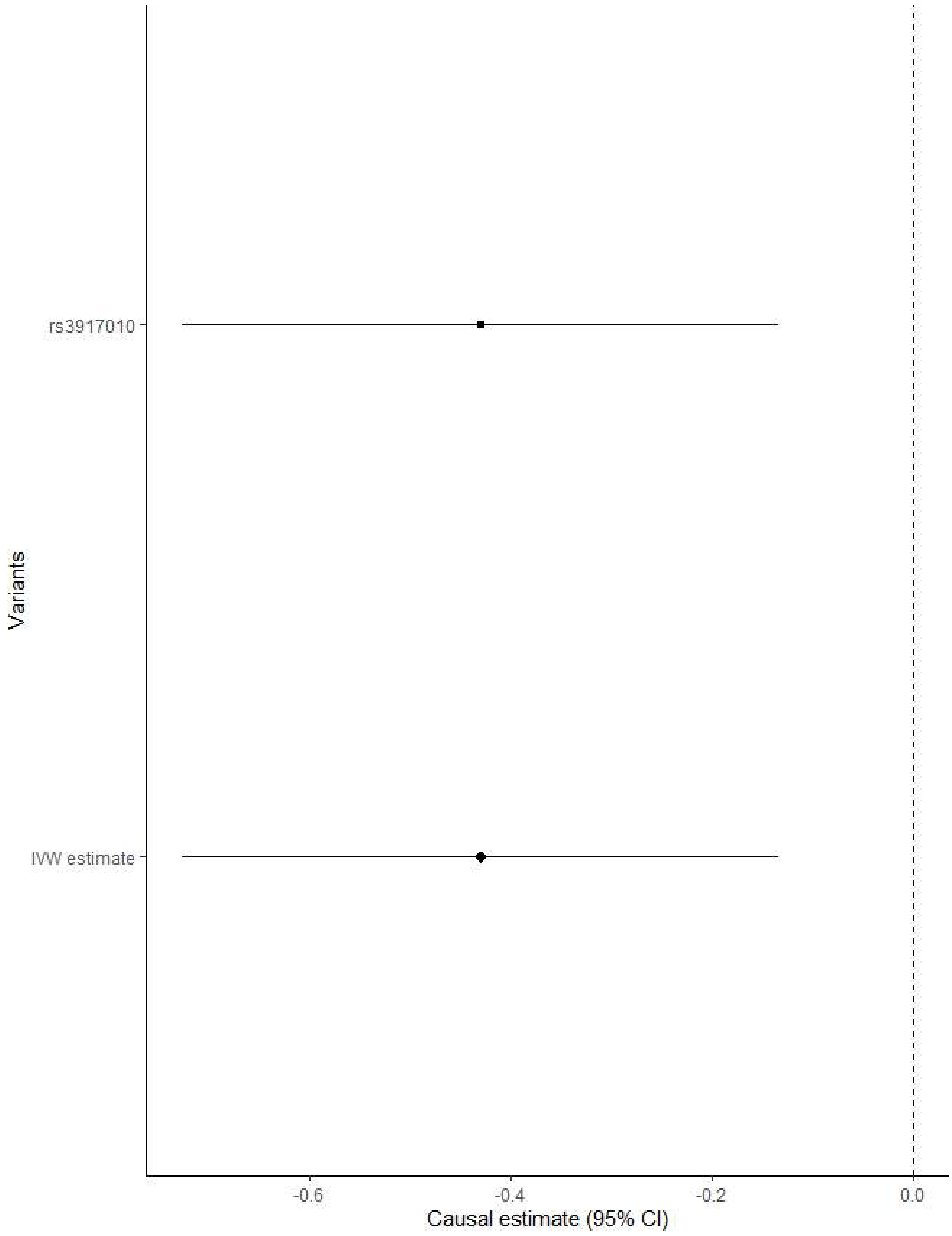

Abbreviations: IVW, Inverse Variance Weighted method; CI, confidence interval; sVCAM, soluble vascular cell adhesion molecule 1.

Figure S15: Iterative leave-one-out analysis for Mendelian randomisation analysis of genetically-proxied TNF-a and atopic asthma, related to STAR methods

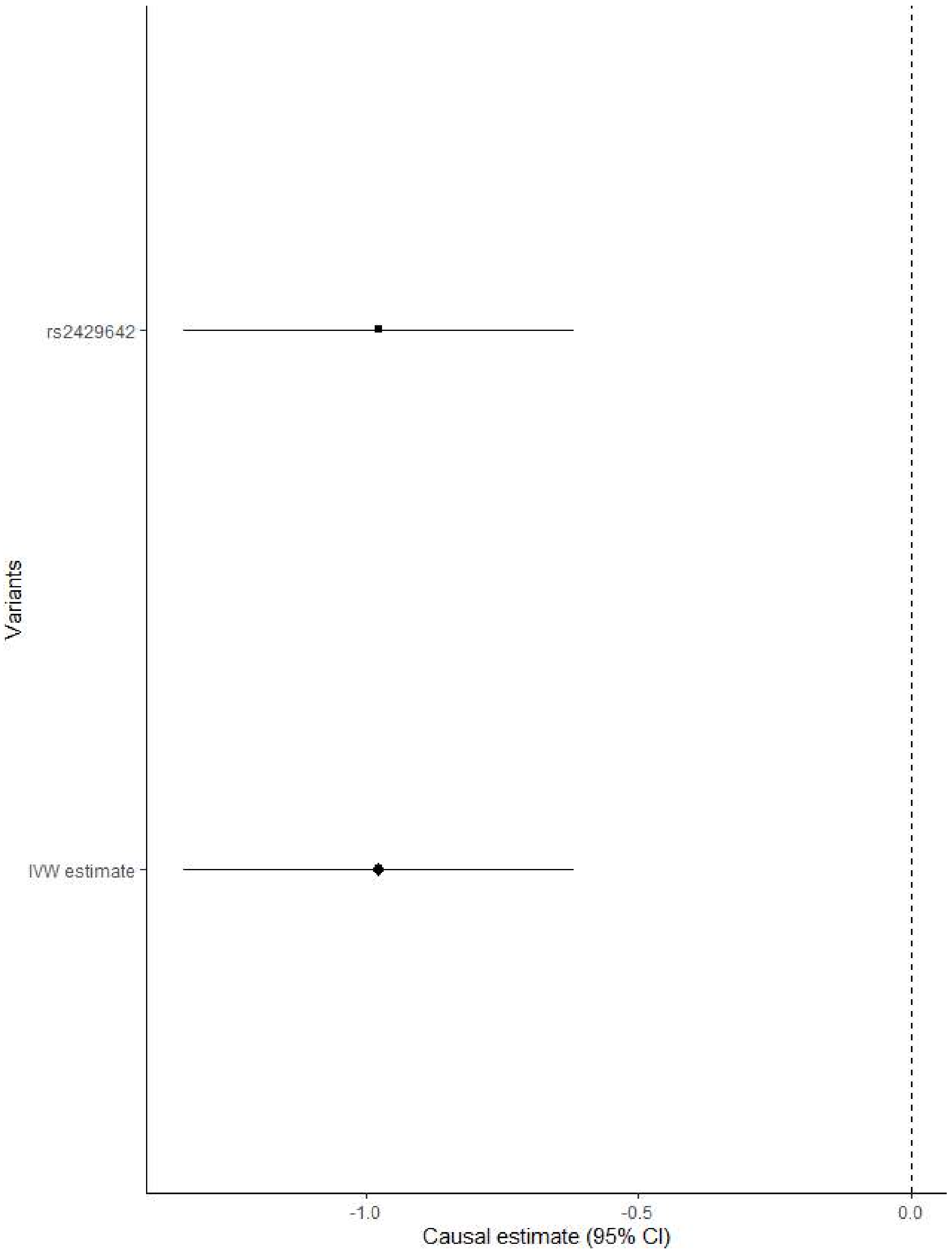

Abbreviations: IVW, Inverse Variance Weighted method; CI, confidence interval; TNF-a, tumor necrosis factor a.

Data S1: STROBE-MR checklist of recommended items to address in reports of Mendelian randomization studies [1,2], related to STAR methods

Item No.	Section		Checklist item	Page No.	Relevant text from manuscript
	**TITLE and ABSTRACT**		Indicate Mendelian randomization (MR) as the study's design in the title and/or the abstract if that is a main purpose of the study		Title - Abstract
	**INTRODUCTION**				
			Explain the scientific background and rationale for the reported study. What is the exposure? Is a potential causal relationship between exposure and outcome plausible? Justify why MR is a helpful method to address the study question		Introduction - paragraphs 1,2
3	**Objectives**		State specific objectives clearly, including pre-specified causal hypotheses (if any). State that MR is a method that, under specific assumptions, intends to estimate causal effects		Introduction - paragraphs 2,3
4	**Study design and data sources**		Present key elements of the study design early in the article. Consider including a table listing sources of data for all phases of the study. For each data source contributing to the analysis, describe the following:		
			Setting: Describe the study design and the underlying population, if possible. Describe the setting, locations, and relevant dates, including periods of recruitment, exposure, follow-up, and data collection, when available.		
		b)	Participants: Give the eligibility criteria, and the sources and methods of selection of participants. Report the sample size, and whether any power or sample size calculations were carried out prior to the main analysis		
			Describe measurement, quality control and selection of genetic variants		
		d)	For each exposure, outcome, and other relevant variables, describe methods of assessment and diagnostic criteria for diseases		STAR Methods
			Provide details of ethics committee approval and participant informed consent, if relevant		Ethics Approval and Consent to Participate
5	**Assumptions**		Explicitly state the three core IV assumptions for the main analysis (relevance, independence and exclusion restriction) as well assumptions for any additional or sensitivity analysis	-	
	**Statistical methods: main analysis**		Describe statistical methods and statistics used		

		a)	Describe how quantitative variables were handled in the analyses (i.e., scale, units, model)	
		b)	Describe how genetic variants were handled in the analyses and, if applicable, how their weights were selected	
		c)	Describe the MR estimator (e.g. two-stage least squares, Wald ratio) and related statistics. Detail the included covariates and, in case of two-sample MR, whether the same covariate set was used for adjustment in the two samples	
		d)	Explain how missing data were addressed	Not applicable
		e)	If applicable, indicate how multiple testing was addressed	
	**Assessment of assumptions**		Describe any methods or prior knowledge used to assess the assumptions or justify their validity	
8	**Sensitivity analyses and additional analyses**		Describe any sensitivity analyses or additional analyses performed (e.g. comparison of effect estimates from different approaches, independent replication, bias analytic techniques, validation of instruments, simulations)	
	**Software and pre-registration**			
		a)	Name statistical software and package(s), including version and settings used	
			State whether the study protocol and details were pre-registered (as well as when and where)	Not applicable
	**RESULTS**			
				N/A
			Report summary statistics for phenotypic exposure(s), outcome(s), and other relevant variables (e.g. means, SDs, proportions)	N/A
				N/A
			For two-sample MR:<br>i. Provide justification of the similarity of the genetic variant-exposure associations between the exposure and outcome samples	N/A

			ii. Provide information on the number of individuals who overlap between the exposure and outcome studies	N/A
11	**Main results**			
		a)	Report the associations between genetic variant and exposure, and between genetic variant and outcome, preferably on an interpretable scale	
		b)	Report MR estimates of the relationship between exposure and outcome, and the measures of uncertainty from the MR analysis, on an interpretable scale, such as odds ratio or relative risk per SD difference	Results, Suppl. tables S2, S3
		c)	If relevant, consider translating estimates of relative risk into absolute risk for a meaningful time period	Not applicable
		d)	Consider plots to visualize results (e.g. forest plot, scatterplot of associations between genetic variants and outcome versus between genetic variants and exposure)	Figures 3, 4
	**Assessment of assumptions**			
			Report any additional statistics (e.g., assessments of heterogeneity across genetic variants, such as I^2, Q statistic or E-value)	
13	**Sensitivity analyses and additional analyses**			
		a)	Report any sensitivity analyses to assess the robustness of the main results to violations of the assumptions	Results, Suppl. Tables S2, S3, S9
		b)	Report results from other sensitivity analyses or additional analyses	
		c)	Report any assessment of direction of causal relationship (e.g., bidirectional MR)	Not applicable
		d)	When relevant, report and compare with estimates from non-MR analyses	
		e)	Consider additional plots to visualize results (e.g., leave-one-out analyses)	Suppl. figures S1-S15
	**DISCUSSION**			
14	**Key results**		Summarize key results with reference to study objectives	Discussion - paragraphs 1, 2, 3
15	**Limitations**		Discuss limitations of the study, taking into account the validity of the IV assumptions, other sources of potential bias, and imprecision. Discuss both direction and magnitude of any potential bias and any efforts to address them	

		a)	Meaning: Give a cautious overall interpretation of results in the context of their limitations and in comparison with other studies	Discussion - paragraph 6
			Mechanism: Discuss underlying biological mechanisms that could drive a potential causal relationship between the investigated exposure and the outcome, and whether the gene-environment equivalence assumption is reasonable. Use causal language carefully, clarifying that IV estimates may provide causal effects only under certain assumptions	Discussion - paragraphs 2, 3
		c)	Clinical relevance: Discuss whether the results have clinical or public policy relevance, and to what extent they inform effect sizes of possible interventions	Discussion - paragraphs 4, 6
			Discuss the generalizability of the study results (a) to other populations, (b) across other exposure periods/timings, and (c) across other levels of exposure	
	**OTHER INFORMATION**			
			Describe sources of funding and the role of funders in the present study and, if applicable, sources of funding for the databases and original study or studies on which the present study is based	Funding
19	**Data and data sharing**		Provide the data used to perform all analyses or report where and how the data can be accessed, and reference these sources in the article. Provide the statistical code needed to reproduce the results in the article, or report whether the code is publicly accessible and if so, where	
	**Conflicts of Interest**		All authors should declare all potential conflicts of interest	Conflicts of interest

This checklist is copyrighted by the Equator Network under the Creative Commons Attribution 3.0 Unported (CC BY 3.0) license.

Data S2: Details regarding instrument selection, COVID-19 outcomes, colocalization and observational analysis, related to STAR methods

Cytokine instrument selection

Linkage disequilibrium (LD) structure was based on the European Population in the 1000 Genomes phase 3 reference panel. The genomic locations were identified using the University of California Santa Cruz (UCSC) Genome Browser (https://genome.ucsc.edu, human genome build 19, accessed on 18th June 2019). Gene expression data were obtained from the GTEx database, which provides a catalog of genetic variants that affect gene expression across multiple tissues, using data from 15,201 RNA-sequencing samples from 49 tissues of 838 post-mortem donors (version 8). [3]

Outcome data for COVID-19 (https://www.covid19hg.org/results/)

We used GWAS data for European ancestry individuals from Release 7 for:
(i) Any **COVID-19**, defined as laboratory-confirmed SARS-CoV-2 infection (RNA and/or serology based), physician diagnosis of COVID-19, or self-report as COVID-19 positive versus population controls (cases = 122,616, controls = 2,475,240),
(ii) **COVID-19 hospitalization**, defined as hospitalization with laboratory-confirmed SARS-CoV-2 infection due to COVID-19-related symptoms versus population controls (cases = 32,519, controls = 2,062,805) and
(iii) **Severe COVID-19**, defined as hospitalization with laboratory-confirmed SARS-CoV-2 infection as the primary reason for admission followed by death or respiratory support versus population controls (cases = 13,769, controls = 1,072,442),
and Release 5 for:
(iv) **Case-only COVID-19 hospitalization**, defined as hospitalization with laboratory-confirmed SARS-CoV-2 infection due to COVID-19-related symptoms versus non-hospitalization with SARS-CoV-2 infection due to COVID-19-related symptoms (cases = 4,829, controls = 11,816).

Colocalization

For each of the cytokine-outcome associations, we used the genomic region extending 50 kb on both sides of the lead cytokine variant. We used a Bayesian framework proposed by Giambartolomei, [4] that calculates posterior probabilities of several causal variant configurations, namely, no causal variant (H0), causal variant for exposure only (H1), causal variant for outcome only (H2), two distinct causal variants (H3) and a common causal variant (H4), under the assumption of a maximum of one causal variant for each trait, and we used a p12 prior (i.e. prior probability that a SNP is associated with both traits) threshold of 5×10^{-04}. Results were considered as supportive of our MR results (i.e., with no evidence of genetic confounding) when PP of H4 was larger than 0.5, or alternatively, when the following two criteria were present: the sum of PP of H4 and PP of H3 was larger than 0.5, and PP of H4 was larger than PP of H3. This algorithm, described in a relevant publication, [5] was used due to low power. When evidence for colocalization was poor and there were multiple potentially causal variants (at least 2 independent instruments) we used the coloc Sum of Single Effects (SuSiE) framework, that relaxes the single causal variant assumption, to identify independent genetic signals and perform pair-wise colocalization analyses on all possible pairs of signals between the traits (the LD structure was accounted for using the European population of the 1000 Genomes phase 3 reference panel). [6]

Observational analysis in the UK Biobank

The associations that emerged in the MR analyses were explored in the UK biobank (UKBB, proposal ID: 79696). [7] The UKBB is an ongoing prospective cohort study which enrolled 502,412 participants aged 40 to 69 years from 22 assessment centers across the UK between 2006 and 2010. [8] At enrollment, participants provided signed consent, and answered questions on socio-demographic, lifestyle and health-related factors, and completed a range of physical measures. They also provided blood, urine and saliva samples, which were subsequently used to perform a range of assays, such as proteomic analyses. Blood plasma samples randomly selected from 54,306 UKBB participants were analyzed using the Olink Explore 1536 platform, capturing 1,463 unique proteins. [9] The following proteins codes were included in the observational analyses: 1392 - Interleukin-1 receptor antagonist protein (IL1ra), 1380 - Interleukin-18 (IL18), 401 - C-C motif chemokine 7 (MCP3), 696 - Macrophage colony-stimulating factor 1 (MCSF), 1314 - Intercellular adhesion molecule 1 (sICAM), 2847 - Vascular cell adhesion protein 1 (sVCAM), 2712 - Tumor necrosis factor (TNFa). Circulating plasma protein concentrations were inverse-rank normalized before modelling.

Participants were followed through record linkage to the National Health Service central registers and death registries. We used ICD-10 code U07.1 in the field IDs 41270, 41270 & 40001 to define severe Covid-19 (Covid-19 hospitalization or death), and the same code in field ID 40001 to define Covid-19 deaths. We used ICD10 codes J43 and J44, in the field IDs 40000, 41202, 41203, 41270, and 41271, to define incident chronic obstructive pulmonary disease (COPD) cases. We used self-reported category 9 (Hayfever, allergic rhinitis or eczema) in the filed ID 6152 (Blood clot, DVT, bronchitis, emphysema, asthma, rhinitis, eczema, allergy diagnosed by doctor) and data field 3786 (Age asthma diagnosed) to define atopic asthma (most cases were prevalent). We used forced vital capacity (FVC) and forced expiratory volume in 1-second (FEV_1) measurements from the baseline assessment (field IDs 3062, and 3063 respectively).

The association of circulating plasma protein concentrations with outcomes was evaluated using regression models, adjusting for age at baseline (continuous), sex (male, female), body mass index (continuous), smoking status (never, former, current), smoking intensity (pack-years of smoking) and smoking status × smoking intensity.

Data S3: Code used for the main analysis (for the outcome "covid vs. population"), related to STAR methods

```
library(ieugwasr)

library(data.table)

library(MendelianRandomization)

library(readr)

eqtl <- fread("Z:/ CYTOKINES MR/cytokines_eQTL.txt")

dim(eqtl)

head(eqtl)

colnames(eqtl)[2]=c("index_rsID")

colnames(eqtl)[5:6]=c("index_A1","index_A2")

colnames(eqtl)[7:9]=c("index_beta","index_se","index_pvalue")

eqtl$ID<-paste0(eqtl$chr_name,":",eqtl$chrom_start)

colnames(eqtl)

eqtl<-eqtl[,c(1,38,19,5:6,35,7:10)]

head(eqtl)

library(R.utils)

outcome<-fread("Z:
/CYTOKINESMR/Outcome/COVID19_HGI_C2v6_ALL_eur_leave_23andme_20210622.b37.txt.gz")

head(outcome)

colnames(outcome)[18:19]=c("REF1","ALT1")

table(outcome$REF==outcome$REF1)

table(outcome$ALT==outcome$ALT1)
```

```
outcome<-outcome[,c(1:17)]

colnames(outcome)[1]="CHR"

outcome$ID<-paste0(outcome$CHR,":",outcome$POS)

sapply(eqtl, function(x) sum(is.na(x)))

sapply(outcome, function(x) sum(is.na(x)))

dim(eqtl)

table(eqtl$cytokine)

x<-unique(eqtl$cytokine)

x

table_ivw<-matrix(NA, nrow=length(x), ncol=22)

colnames(table_ivw)=c("Cytokine","IVW  Estimate","IVW CILower","IVW CIUpper","IVW_Pvalue","N of SNPs","Simple median Estimate","Simple median CILower","Simple median CIUpper","Simple median Pvalue","Weighted median Estimate","Weighted median CILower","Weighted median CIUpper","Weighted median Pvalue","MR Egger Estimate","MR Egger CILower","MR Egger CIUpper","MR Egger Pvalue","MR Egger (intercept) Estimate","MR Egger (intercept) CILower","MR Egger (intercept) CIUpper","MR Egger (intercept) Pvalue")

table_ivw

for (i in (1:length(x))) {

   cytt<-assign(paste("eqtl",i,sep="."), eqtl[eqtl$cytokine==x[i],])

   eqtl.outcome.merge=merge(cytt, outcome, by="ID")

   dim(eqtl.outcome.merge)

   head(eqtl.outcome.merge)
```

```
colnames(eqtl.outcome.merge)[c(13:14,24)] =
c("outcome_A2","outcome_A1","outcome_EAF_not_alligned")

colnames(eqtl.outcome.merge)[17:19] =
c("outcome_beta_not_alligned","outcome_se","outcome_pvalue")

head(eqtl.outcome.merge)

eqtl.outcome.merge$index_A1<-tolower(eqtl.outcome.merge$index_A1)

eqtl.outcome.merge$index_A2<-tolower(eqtl.outcome.merge$index_A2)

eqtl.outcome.merge$outcome_A1<-tolower(eqtl.outcome.merge$outcome_A1)

eqtl.outcome.merge$outcome_A2<-tolower(eqtl.outcome.merge$outcome_A2)

table(eqtl.outcome.merge$index_A1 == eqtl.outcome.merge$outcome_A1)

table(eqtl.outcome.merge$index_A1 == eqtl.outcome.merge$outcome_A2)

inconsistent=which((eqtl.outcome.merge$index_A1 != eqtl.outcome.merge$outcome_A1)

               & (eqtl.outcome.merge$index_A1 != eqtl.outcome.merge$outcome_A2))

eqtl.outcome.merge[inconsistent,]

dim(eqtl.outcome.merge)

if (nrow(eqtl.outcome.merge[inconsistent,])!=0) {

  eqtl.outcome.merge = eqtl.outcome.merge[-inconsistent,]

}

dim(eqtl.outcome.merge)

table(eqtl.outcome.merge$index_A2 == eqtl.outcome.merge$outcome_A1)

table(eqtl.outcome.merge$index_A2 == eqtl.outcome.merge$outcome_A2)

inconsistent2=which((eqtl.outcome.merge$index_A2 != eqtl.outcome.merge$outcome_A1)
```

```
                    & (eqtl.outcome.merge$index_A2 != eqtl.outcome.merge$outcome_A2))
    eqtl.outcome.merge[inconsistent2,]
    dim(eqtl.outcome.merge)
    if (nrow(eqtl.outcome.merge[inconsistent2,])!=0) {
      eqtl.outcome.merge = eqtl.outcome.merge[-inconsistent2,]
    }
    dim(eqtl.outcome.merge)
    eqtl.outcome.merge$outcome_beta = ifelse(eqtl.outcome.merge$index_A1 ==
eqtl.outcome.merge$outcome_A1,
eqtl.outcome.merge$outcome_beta_not_alligned, -1*eqtl.outcome.merge$outcome_beta_not_alligned)


    eqtl.outcome.merge$outcome_EAF = ifelse(eqtl.outcome.merge$index_A1 ==
eqtl.outcome.merge$outcome_A1, eqtl.outcome.merge$outcome_EAF_not_alligned, 1-
eqtl.outcome.merge$outcome_EAF_not_alligned)
    colnames(eqtl.outcome.merge)[2]="rsid"
    colnames(eqtl.outcome.merge)[9]="pval"
    head(eqtl.outcome.merge)
    eqtl.outcome.clump = ieugwasr::ld_clump(eqtl.outcome.merge, clump_r2=0.1)
    dim(eqtl.outcome.clump)
    head(eqtl.outcome.clump)
    sum(duplicated(eqtl.outcome.clump$rsid))
     eqtl.outcome.clump <- eqtl.outcome.clump[!duplicated(eqtl.outcome.clump$rsid), ]
    head(eqtl.outcome.clump)
```

```
rs.id = eqtl.outcome.clump$rsid
index_beta = eqtl.outcome.clump$index_beta
index_se = eqtl.outcome.clump$index_se
outcome_beta = eqtl.outcome.clump$outcome_beta
outcome_se = eqtl.outcome.clump$outcome_se
mr.input = mr_input(bx = index_beta, bxse = index_se, by = outcome_beta, byse = outcome_se,
                exposure = "cytokine", outcome = "Outcome", snps = rs.id)
ac<-mr_ivw(mr.input)
table_ivw[i,1]<-x[i]
table_ivw[i,2]<-ac$Estimate
table_ivw[i,3]<-ac$CILower
table_ivw[i,4]<-ac$CIUpper
table_ivw[i,5]<-ac$Pvalue
table_ivw[i,6]<-ac$SNPs
 if (ac$SNPs>2){
  ab<-mr_allmethods(mr.input)
  table_ivw[i,7]<-ab$Values[1,2]
  table_ivw[i,8]<-ab$Values[1,4]
  table_ivw[i,9]<-ab$Values[1,5]
  table_ivw[i,10]<-ab$Values[1,6]
  table_ivw[i,11]<-ab$Values[2,2]
  table_ivw[i,12]<-ab$Values[2,4]
```

```
        table_ivw[i,13]<-ab$Values[2,5]

        table_ivw[i,14]<-ab$Values[2,6]

        table_ivw[i,15]<-ab$Values[8,2]

        table_ivw[i,16]<-ab$Values[8,4]

        table_ivw[i,17]<-ab$Values[8,5]

        table_ivw[i,18]<-ab$Values[8,6]

        table_ivw[i,19]<-ab$Values[9,2]

        table_ivw[i,20]<-ab$Values[9,4]

        table_ivw[i,21]<-ab$Values[9,5]

        table_ivw[i,22]<-ab$Values[9,6]


      }


  }
```

References

1. Skrivankova, V. W., Richmond, R. C., Woolf, B. A. R., Davies, N. M., Swanson, S. A., VanderWeele, T. J., Timpson, N. J., Higgins, J. P. T., Dimou, N., Langenberg, C., et al. (2021). Strengthening the reporting of observational studies in epidemiology using mendelian randomisation (STROBE-MR): explanation and elaboration. BMJ (Clinical research ed.), 375, n2233. https://doi.org/10.1136/bmj.n2233
2. Skrivankova, V. W., Richmond, R. C., Woolf, B. A. R., Yarmolinsky, J., Davies, N. M., Swanson, S. A., VanderWeele, T. J., Higgins, J. P. T., Timpson, N. J., Dimou, N., et al. (2021). Strengthening the Reporting of Observational Studies in Epidemiology Using Mendelian Randomization: The STROBE-MR Statement. JAMA, 326(16), 1614–1621. https://doi.org/10.1001/jama.2021.18236
3. GTEx Consortium (2020). The GTEx Consortium atlas of genetic regulatory effects across human tissues. Science (New York, N.Y.), 369(6509), 1318–1330. https://doi.org/10.1126/science.aaz1776
4. Giambartolomei, C., Vukcevic, D., Schadt, E. E., Franke, L., Hingorani, A. D., Wallace, C., & Plagnol, V. (2014). Bayesian test for colocalisation between pairs of genetic association studies using summary statistics. PLoS genetics, 10(5), e1004383. https://doi.org/10.1371/journal.pgen.1004383
5. Zuber, V., Grinberg, N. F., Gill, D., Manipur, I., Slob, E. A. W., Patel, A., Wallace, C., & Burgess, S. (2022). Combining evidence from Mendelian randomization and colocalization: Review and comparison of approaches. American journal of human genetics, 109(5), 767–782. https://doi.org/10.1016/j.ajhg.2022.04.001
6. Wallace C. (2021). A more accurate method for colocalisation analysis allowing for multiple causal variants. PLoS genetics, 17(9), e1009440. https://doi.org/10.1371/journal.pgen.1009440
7. UK Biobank, https://www.ukbiobank.ac.uk/
8. Sudlow, C., Gallacher, J., Allen, N., Beral, V., Burton, P., Danesh, J., Downey, P., Elliott, P., Green, J., Landray, M., et al. (2015). UK biobank: an open access resource for identifying the causes of a wide range of complex diseases of middle and old age. PLoS medicine, 12(3), e1001779. https://doi.org/10.1371/journal.pmed.1001779
9. Sun, B.B., Chiou, J., Traylor, M., Benner, C., Hsu, Y.H., Richardson, T.G., Surendran, P., Mahajan, A., Robins, C., Vasquez-Grinnell, S.G., et al. (2022). Genetic regulation of the human plasma proteome in 54,306 UK Biobank participants. Available from bioRxiv: https://doi.org/10.1101/2022.06.17.496443
